# Supplementary material for: A fast non-parametric test of association for multiple traits
Source: Genome Biol. 2023 Oct 12;24:230. doi: 10.1186/s13059-023-03076-8 (PMC10571397; doi:10.1186/s13059-023-03076-8)
Supplement: Supplementary file 1 — Additional file 1. Supplementary Tables, Figures and Notes. [file 13059_2023_3076_MOESM1_ESM.pdf]

Supplementary Tables, Figures and Notes

A fast non-parametric test of association for multiple traits

Diego Garrido-Martín*[1,2], Miquel Calvo[1], Ferran Reverter[1] and Roderic Guigó[2,3]

[1]Department of Genetics, Microbiology and Statistics, Universitat de Barcelona (UB), Av. Diagonal 643, Barcelona 08028, Spain

[2]Centre for Genomic Regulation (CRG), The Barcelona Institute of Science and Technology, Dr. Aiguader 88, Barcelona 08003, Catalonia, Spain

[3]Universitat Pompeu Fabra (UPF), Barcelona, Catalonia, Spain

*Correspondence should be addressed to e-mail: dgarrido@ub.edu (Diego Garrido-Martín).

Tissue	EA	AA	Variants	Genes	pb-sQTLs	pb-sGenes
Adipose - Subcutaneous	492	71	325,445	9,697	1,277	176
Adipose - Visceral (Omentum)	402	51	162,236	8,618	197	30
Adrenal Gland	200	25	5,855	1,462	0	0
Artery - Aorta	338	38	45,281	5,455	12	6
Artery - Coronary	180	25	6,219	1,553	0	0
Artery - Tibial	489	76	345,493	9,464	1,114	139
Breast - Mammary Tissue	337	47	123,365	8,004	160	34
Cells - Cultured fibroblasts	416	58	189,829	8,142	331	56
Cells - EBV-transformed lymphocytes	116	29	6,753	1,386	6	1
Colon - Sigmoid	273	34	22,573	3,751	17	6
Colon - Transverse	305	50	141,357	8,100	97	28
Esophagus - Gastroesophageal Junction	281	36	33,299	4,538	10	4
Esophagus - Mucosa	424	60	223,860	9,237	473	69
Esophagus - Muscularis	396	55	206,883	9,056	195	32
Heart - Atrial Appendage	322	42	70,406	6,333	19	6
Heart - Left Ventricle	334	42	53,526	5,338	36	2
Lung	444	58	241,674	9,871	385	47
Minor Salivary Gland	118	23	4,224	1,129	5	3
Muscle - Skeletal	602	86	327,757	8,439	1,499	199
Nerve - Tibial	449	67	329,639	10,187	619	108
Ovary	140	21	2,298	730	0	0
Pancreas	252	39	40,302	4,908	5	4
Prostate	186	27	13,958	2,171	57	3
Skin - Not Sun Exposed (Suprapubic)	440	62	249,241	9,648	602	100
Skin - Sun Exposed (Lower leg)	518	73	333,619	10,090	1,938	270
Small Intestine - Terminal Ileum	144	25	5,919	1,509	0	0
Spleen	185	34	20,486	3,638	6	3
Stomach	269	44	80,945	6,776	14	7
Testis	277	34	40,283	5,036	5	5
Thyroid	494	62	284,363	10,085	616	105
Whole Blood	574	80	155,988	5,664	555	60
				**Total (unique)**	7,719	938

Table S1. Number of individuals of each ancestry (European American, EA, and African American, AA), variants and genes tested; pb-sQTLs and pb-sGenes (genes with at least one pb-sQTL) identified across tissues after multiple testing correction (see Methods).

	European American			African American		
	**AA**	**AC**	**CC**	**AA**	**AC**	**CC**
I1 in *KLK5-203* (chr19:50,952,668-50,952,950)	82 (0.26)	87 (0.32)	108 (0.43)	90 (0.30)	111 (0.28)	127 (0.32)
I2 in *KLK5-202* (chr19:50,952,814-50,952,950)	29 (0.09)	25 (0.09)	18 (0.07)	26 (0.09)	40 (0.10)	37 (0.09)
I3 in *KLK5-201* and *KLK5-202* (chr19:50,952,668-50,952,747)	206 (0.65)	160 (0.59)	126 (0.50)	180 (0.61)	242 (0.62)	238 (0.59)

Table S2. Median number (proportion) of reads supporting three alternative *KLK5* 5' UTR introns, across individuals with different ancestries (European American, African American) and genotypes at rs2739412 (chr19:50,952,558, A/C).

Locus	chr	start	end	Lead SNP	Position	*p* value
1	1	43,760,236	43,949,718	rs1004291	43,858,630	$8.67 \cdot 10^{-11}$
2	1	47,974,123	47,980,916	rs6658111	47,980,916	$2.82 \cdot 10^{-9}$
3	1	50,828,316	51,480,258	rs11584472	51,114,503	$1.83 \cdot 10^{-8}$
4	1	155,033,308	155,087,188	rs11589479	155,033,308	$1.02 \cdot 10^{-14}$
5	2	134,241,198	134,533,762	rs3936142	134,471,302	$3.39 \cdot 10^{-9}$
6	2	162,796,517	162,891,848	rs2909455	162,843,078	$5.92 \cdot 10^{-14}$
7	3	55,535,699	55,560,144	rs73077403	55,558,889	$3.19 \cdot 10^{-9}$
8	3	55,943,930	56,295,551	rs17825124	56,052,060	$1.23 \cdot 10^{-8}$
9	3	190,591,315	190,836,742	rs967512	190,664,073	$1.00 \cdot 10^{-14}$
10	4	20,088,468	20,239,195	rs13150272	20,121,121	$9.01 \cdot 10^{-11}$
11	4	187,366,735	187,390,722	4:187389308_TA_T	187,389,308	$3.15 \cdot 10^{-12}$
12	5	65,923,428	66,219,202	rs57246240	66,112,715	$1.00 \cdot 10^{-14}$
13	5	90,796,839	91,040,975	rs6865573	90,806,861	$1.00 \cdot 10^{-14}$
14	5	92,182,752	92,361,481	rs888814	92,186,429	$1.98 \cdot 10^{-10}$
15	5	92,683,607	93,537,741	5:92797041_TG_T	92,797,041	$4.36 \cdot 10^{-11}$
16	6	25,786,226	27,730,334	rs34073492	26,739,452	$4.10 \cdot 10^{-8}$
17	6	146,976,037	147,008,552	rs9497602	146,990,571	$2.35 \cdot 10^{-9}$
18	6	148,041,598	148,095,291	rs9399619	148,056,480	$2.55 \cdot 10^{-9}$
19	7	132,045,957	132,141,199	rs7803579	132,087,061	$5.72 \cdot 10^{-9}$
20	7	155,797,978	155,808,233	rs56354640	155,799,049	$3.52 \cdot 10^{-12}$
21	8	116,464,988	116,636,719	rs2049867	116,588,570	$3.43 \cdot 10^{-10}$
22	9	3,924,447	3,944,778	rs7861718	3,924,447	$5.92 \cdot 10^{-9}$
23	9	18,006,202	18,109,156	rs12337268	18,086,410	$5.80 \cdot 10^{-12}$
24	9	98,216,876	98,280,656	rs28536201	98,276,753	$8.57 \cdot 10^{-9}$
25	9	113,651,047	113,679,463	rs4246883	113,674,955	$1.51 \cdot 10^{-8}$
26	10	50,210,710	50,381,231	rs17700388	50,218,092	$4.36 \cdot 10^{-8}$
27	10	126,302,936	126,564,695	rs4962694	126,436,717	$1.00 \cdot 10^{-14}$
28	11	110,945,939	111,083,785	rs58066679	111,056,158	$3.99 \cdot 10^{-10}$
29	12	65,372,996	66,065,136	rs61921502	65,832,468	$1.00 \cdot 10^{-14}$
30	12	109,029,333	109,260,895	rs7484851	109,134,193	$5.10 \cdot 10^{-11}$
31	12	117,309,440	117,338,504	rs146607495	117,319,202	$1.52 \cdot 10^{-12}$
32	13	81,164,609	81,468,042	rs9545540	81,298,245	$3.12 \cdot 10^{-12}$
33	14	59,064,730	59,092,075	rs186347	59,072,226	$1.00 \cdot 10^{-14}$
34	14	59,629,611	59,804,999	rs1252992	59,665,286	$4.33 \cdot 10^{-12}$
35	15	63,274,450	63,297,719	rs4775593	63,295,172	$4.98 \cdot 10^{-13}$
36	15	86,004,091	86,079,115	rs1531854	86,022,084	$2.17 \cdot 10^{-8}$
37	15	98,429,818	98,444,934	rs5814830	98,431,818	$4.62 \cdot 10^{-11}$
38	15	101,200,873	101,210,973	rs8036545	101,210,641	$3.64 \cdot 10^{-8}$
39	15	101,713,433	101,794,473	15:101778449_ACCAT_A	101,778,449	$4.74 \cdot 10^{-9}$
40	16	70,658,224	70,692,574	rs68049363	70,662,480	$4.28 \cdot 10^{-10}$
41	17	73,439,428	73,532,213	rs77001351	73,498,652	$5.26 \cdot 10^{-9}$

Table S3. Genome-wide significant loci associated with hippocampal subfield volumes, identified by MANTA. Locus definition was performed by the FUMA platform (default parameters). Coordinates correspond to the hg19/GRCh37 human reference genome assembly.

Method	Locus	chr	start	end	Lead SNP	Position	*p* value
MANTA	1	3	190,591,418	190,678,438	rs13079050	190,591,418	$4.22 \cdot 10^{-8}$
	2	5	66,024,969	66,179,049	rs17214309	66,104,105	$4.85 \cdot 10^{-9}$
	3	12	65,718,299	65,905,126	rs79487293	65,905,126	$3.32 \cdot 10^{-9}$
GEMMA	1	3	190,591,418	190,678,743	rs193250936	190,640,759	$1.42 \cdot 10^{-12}$
	2	10	78,204,376	78,643,886	rs10824440	78,489,079	$5.50 \cdot 10^{-9}$
	3	12	65,718,299	65,874,956	rs17178006	65,718,299	$2.62 \cdot 10^{-8}$

Table S4. Genome-wide significant loci associated with hippocampal subfield volumes, identified by MANTA and GEMMA in the downsampled dataset (n = 10,000). Locus definition was performed by the FUMA platform (default parameters). Coordinates correspond to the hg19/GRCh37 human reference genome assembly.

Data-Field	Description
31	Sex
21022	Age at recruitment
22828	Imputation from genotype (WTCHG)
26620	Volume of Hippocampal-tail (left hemisphere)
26621	Volume of subiculum-body (left hemisphere)
26622	Volume of CA1-body (left hemisphere)
26623	Volume of subiculum-head (left hemisphere)
26624	Volume of hippocampal-fissure (left hemisphere)
26625	Volume of presubiculum-head (left hemisphere)
26626	Volume of CA1-head (left hemisphere)
26627	Volume of presubiculum-body (left hemisphere)
26628	Volume of parasubiculum (left hemisphere)
26629	Volume of molecular-layer-HP-head (left hemisphere)
26630	Volume of molecular-layer-HP-body (left hemisphere)
26631	Volume of GC-ML-DG-head (left hemisphere)
26632	Volume of CA3-body (left hemisphere)
26633	Volume of GC-ML-DG-body (left hemisphere)
26634	Volume of CA4-head (left hemisphere)
26635	Volume of CA4-body (left hemisphere)
26636	Volume of fimbria (left hemisphere)
26637	Volume of CA3-head (left hemisphere)
26638	Volume of HATA (left hemisphere)
26639	Volume of Whole-hippocampal-body (left hemisphere)
26640	Volume of Whole-hippocampal-head (left hemisphere)
26641	Volume of Whole-hippocampus (left hemisphere)
26642	Volume of Hippocampal-tail (right hemisphere)
26643	Volume of subiculum-body (right hemisphere)
26644	Volume of CA1-body (right hemisphere)
26645	Volume of subiculum-head (right hemisphere)
26646	Volume of hippocampal-fissure (right hemisphere)
26647	Volume of presubiculum-head (right hemisphere)
26648	Volume of CA1-head (right hemisphere)
26649	Volume of presubiculum-body (right hemisphere)
26650	Volume of parasubiculum (right hemisphere)
26651	Volume of molecular-layer-HP-head (right hemisphere)
26652	Volume of molecular-layer-HP-body (right hemisphere)
26653	Volume of GC-ML-DG-head (right hemisphere)
26654	Volume of CA3-body (right hemisphere)
26655	Volume of GC-ML-DG-body (right hemisphere)
26656	Volume of CA4-head (right hemisphere)
26657	Volume of CA4-body (right hemisphere)
26658	Volume of fimbria (right hemisphere)
26659	Volume of CA3-head (right hemisphere)
26660	Volume of HATA (right hemisphere)
26661	Volume of Whole-hippocampal-body (right hemisphere)
26662	Volume of Whole-hippocampal-head (right hemisphere)
26663	Volume of Whole-hippocampus (right hemisphere)

Table S5. List of Data-Fields retrieved from the UK Biobank. Further details on each Data-Field are available at the UK Biobank data showcase (`https://biobank.ndph.ox.ac.uk/showcase`).

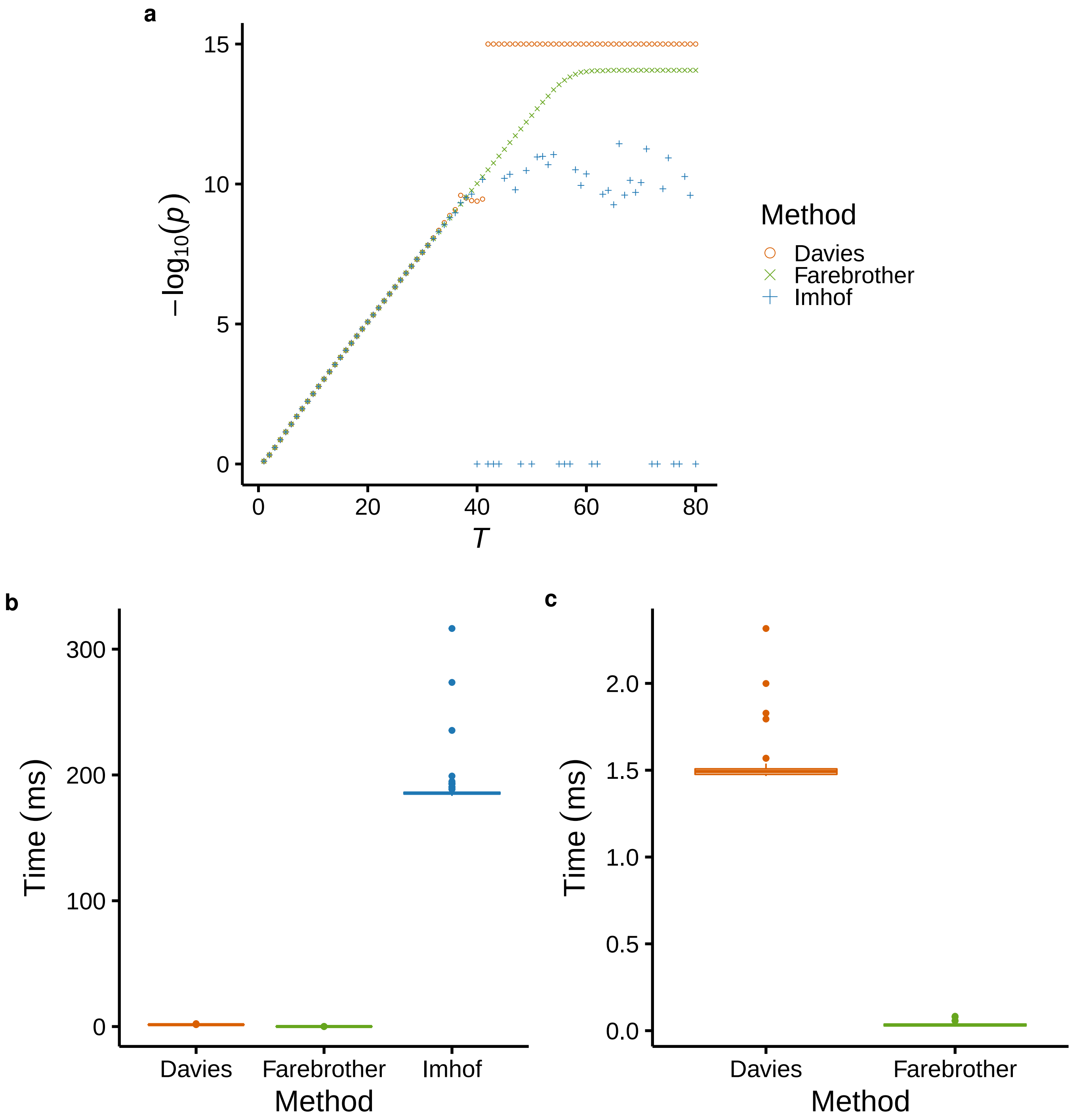

Fig. S1. a) Behaviour of the *p* values obtained by Davies, Farebrother and Imhof algorithms, as implemented in the CompQuadForm R package[1], as a function of different values of the test statistic (labelled as T, x-axis). The data shown correspond to a simulation in which $q = 5$ weights are sampled from a uniform distribution ($\lambda_j \sim U(a = 0, b = 1)$, $j \in \{1, \ldots, 5\}$), $T \in \{1, 2, \ldots, 80\}$, and chi-square variables have 1 degree of freedom. While Farebrother *p* values decrease monotonically with the value of T, down to the precision limit ($\approx 10^{-14}$), Imhof and Davies generated *p* values of 0 (here displayed as $p = 10^{-15}$) or even negative (here displayed as $p = 1$). **b)** Running time of the three methods (in milliseconds), computed across 100 runs with $T = 30$ using the microbenchmark R package[2]. **c)** Zoom of b).

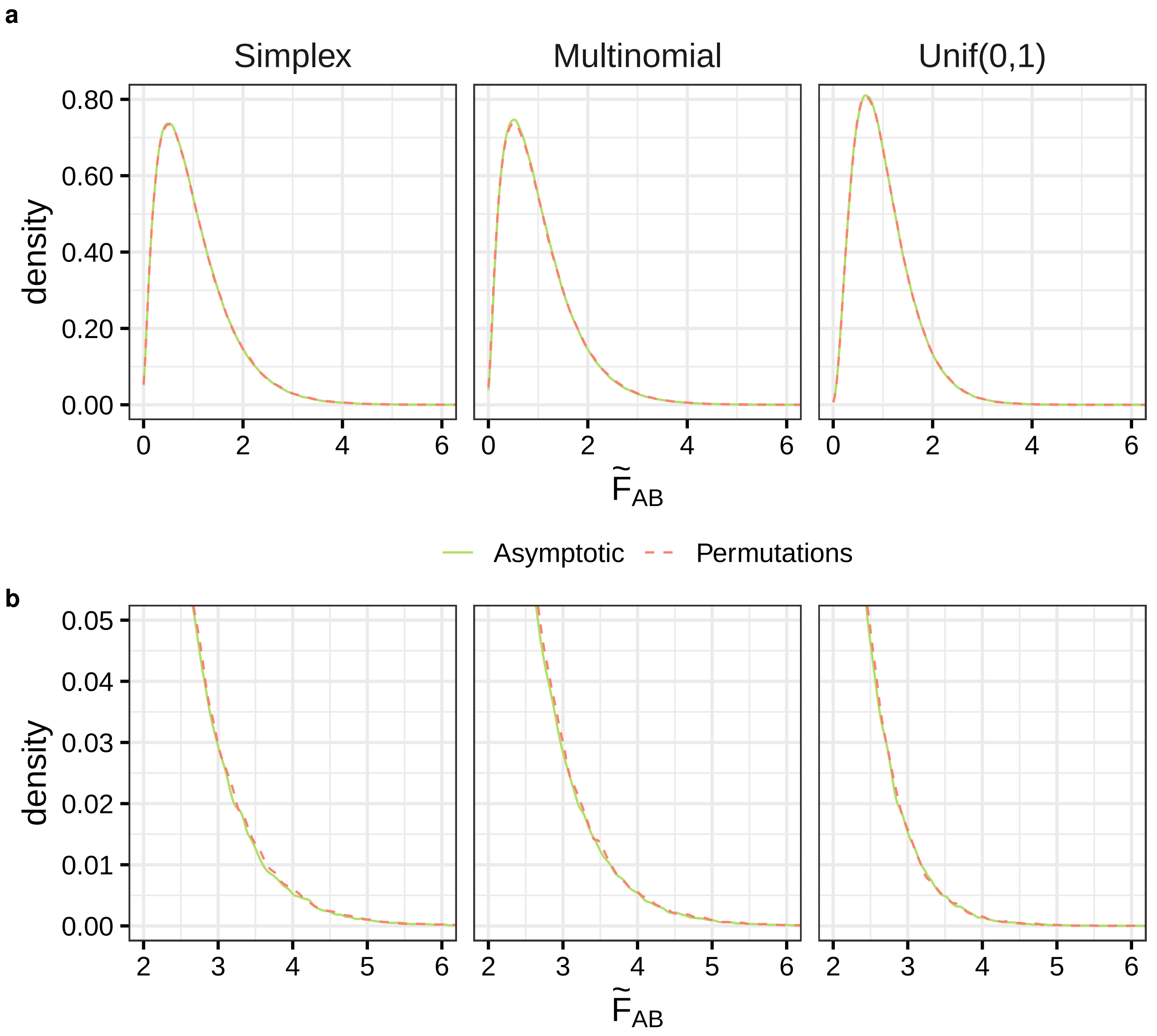

Fig. S2. **a)** Asymptotic null distribution of the PERMANOVA test statistic for the interaction term (green solid line), compared to the empirical null distribution obtained using 10^6 permutations (red dashed line), for different distributions of the response variables: multivariate proportions (left), multinomial (middle), and Gaussian copula with uniform marginals (right). **b)** Zoom of the upper tail of the distribution. Simulation details: model (5), $n = 300$ observations of $q = 3$ response variables, with B generated under H_1, Δ set to 0.1 (simplex, multinomial) and 1 (Gaussian copula) (see Methods).

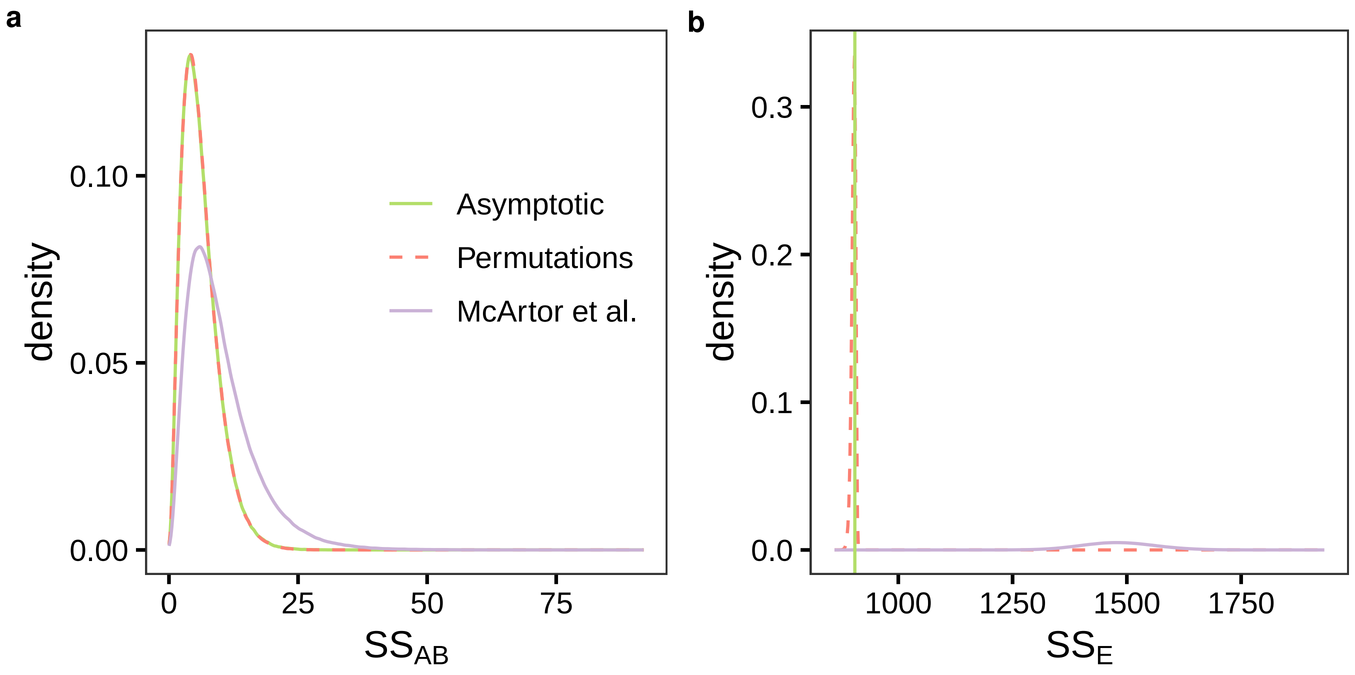

Fig. S3. Null distribution of **a)** the numerator (SS_{AB}) and **b)** the denominator (SS_E) sums of squares of the PERMANOVA test statistic (see equation (2) in Results). We simulated a scenario analogous to the one employed for Fig. 1a (see Results) and studied the test statistic for the interaction term (AB). We compared our asymptotic distribution derived from equation (3) in Results (green solid line), with the proposal of McArtor et al.[3] (purple solid line) and the distribution obtained using 10^6 permutations (red dashed line).

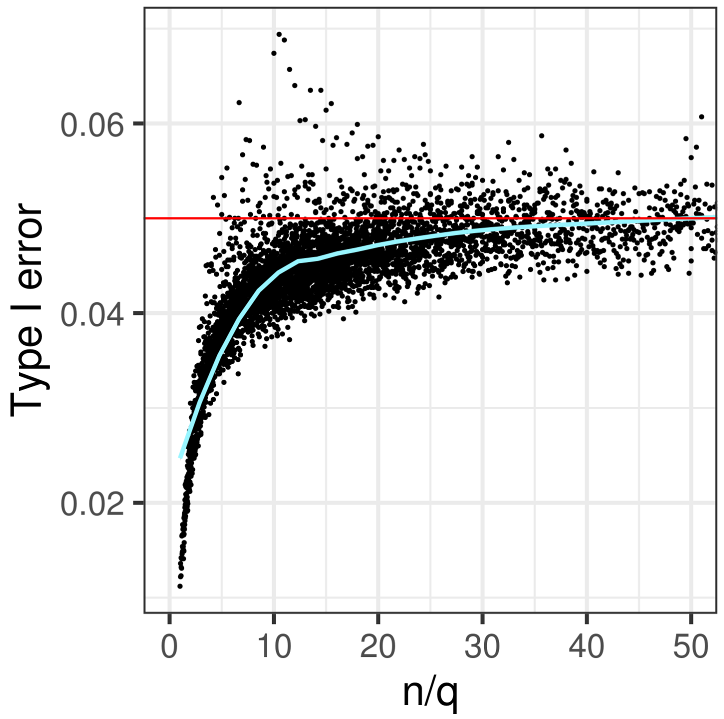

Fig. S4. Type I error of asymptotic *p* values for the interaction term (AB) as a function of the n/q ratio. We considered values of n ranging from 20 to 300, and values of q ranging from 2 to 20. For visualization purposes, we show the window $n/q \in$ [0,50]. Simulation details: $\boldsymbol{y} \sim \mathcal{N}(\mathbf{0}, \mathbf{I}_q)$, model (5) with factor B simulated under H_1 and $\Delta = 1$ (see Methods). The horizontal red line marks the 0. A polynomial has been fitted to the points using local fitting (LOESS), in order to describe the trend (fit shown in blue, 95% confidence interval in gray).

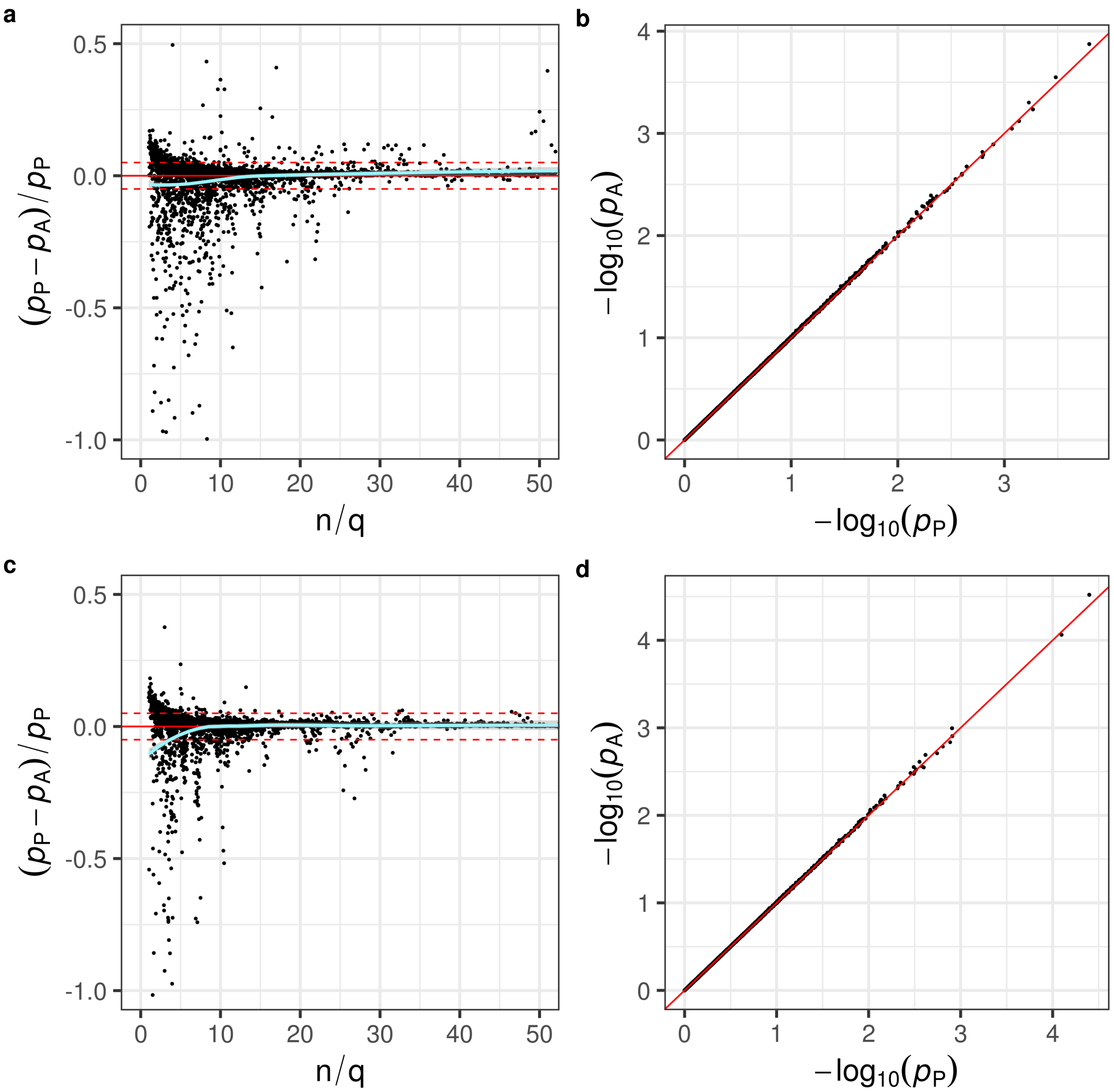

Fig. S5. a, c) Relative bias of asymptotic *p* values *vs* n/q ratio, for different distributions of the response variables: multivariate proportions (simplex) and Gaussian copula with uniform marginals, respectively. Relative difference between asymptotic (p_A) and permutation-based (p_P, 10^5 permutations) *p* values for the interaction term (AB) as a function of the ratio between the total sample size and the number of dependent variables (n/q). We considered values of n ranging from 20 to 300, and values of q ranging from 2 to 20. For visualization purposes, we show values of $n/q \in$ [0,50] and relative biases $\in$ [-1,0.5]. The horizontal solid red line marks the 0. The horizontal dashed red lines mark the 5% relative bias. A polynomial has been fitted to the points using local fitting (LOESS), in order to describe the trend (fit shown in green, 95% confidence interval in gray). **b, d)** Comparison of asymptotic and permutation-based *p* values when the asymptotic null holds ($n = 300$, $q = 3$), for different distributions of the response variables: multivariate proportions (simplex) and Gaussian copula with uniform marginals, respectively. Simulation details: B generated under H_1, with Δ set to 0.02 (simplex) or 1 (Gaussian copula).

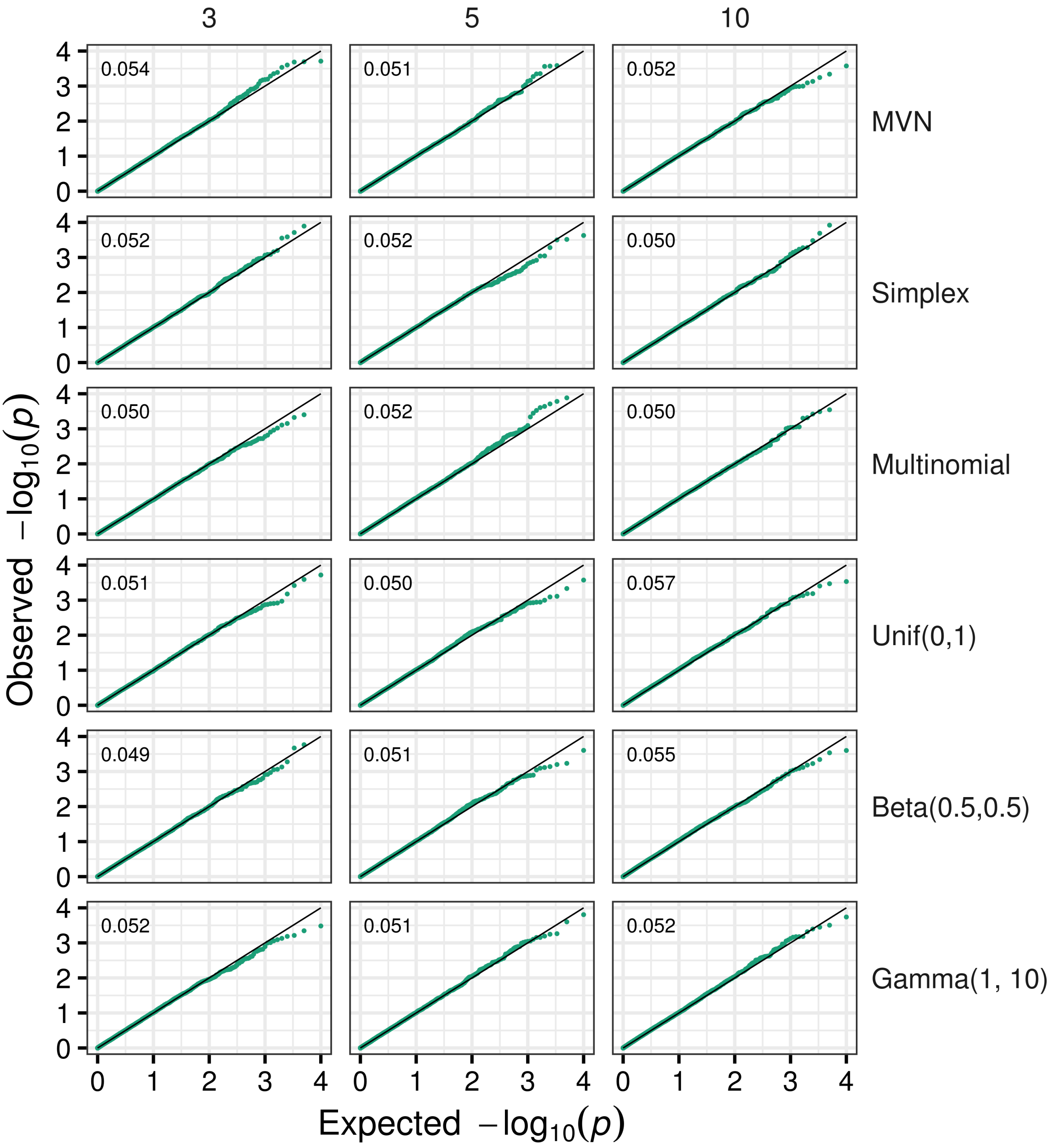

Fig. S6. QQ-plots of *p* values obtained with MANTA in a cohort of unrelated individuals ($n = 1000$), across different numbers of traits (columns) and residual distributions (rows) (see Methods). Type I errors are also shown. In all simulations, h_g^2 was set to 0.2.

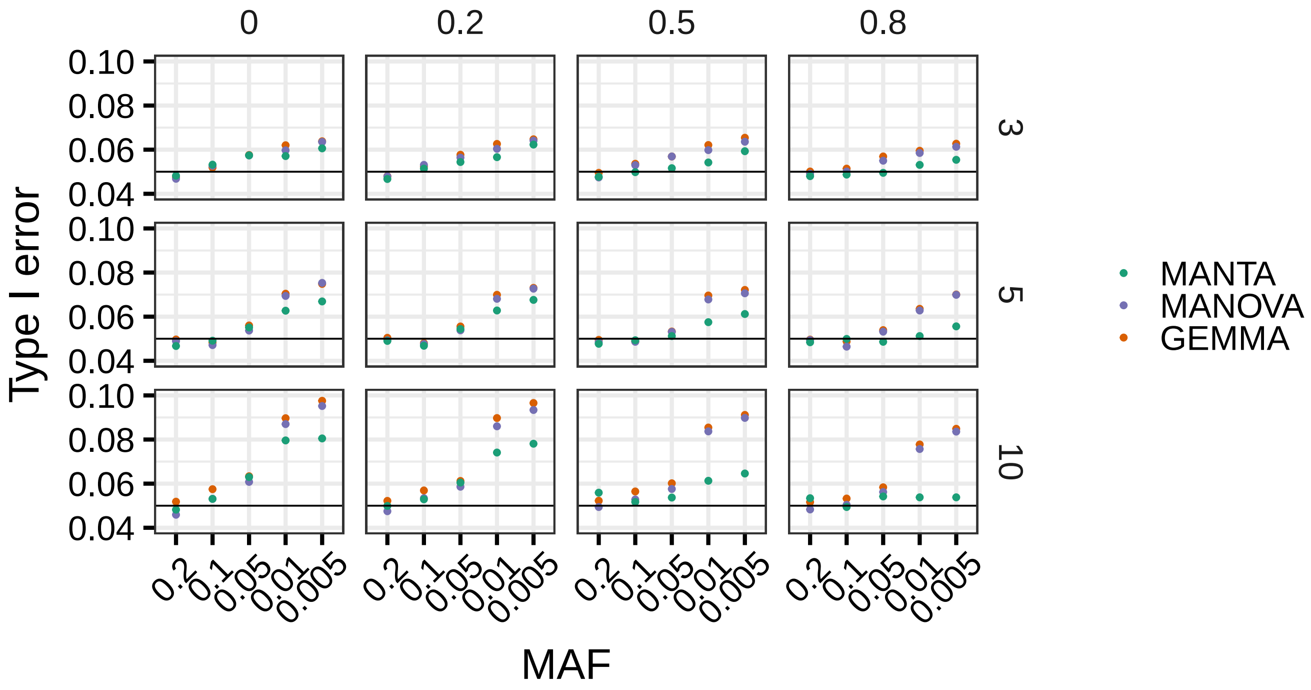

Fig. S7. a). Type I error of MANTA (green), MANOVA (purple) and GEMMA (orange) as a function of the SNP minimum allele frequency (MAF), across different numbers of traits (rows) and pairwise trait correlations (columns). Simulation details: $n = 1000$, $h_g^2 = 0.2$, multivariate t residuals (3 degrees of freedom), binomial SNPs (see Methods).

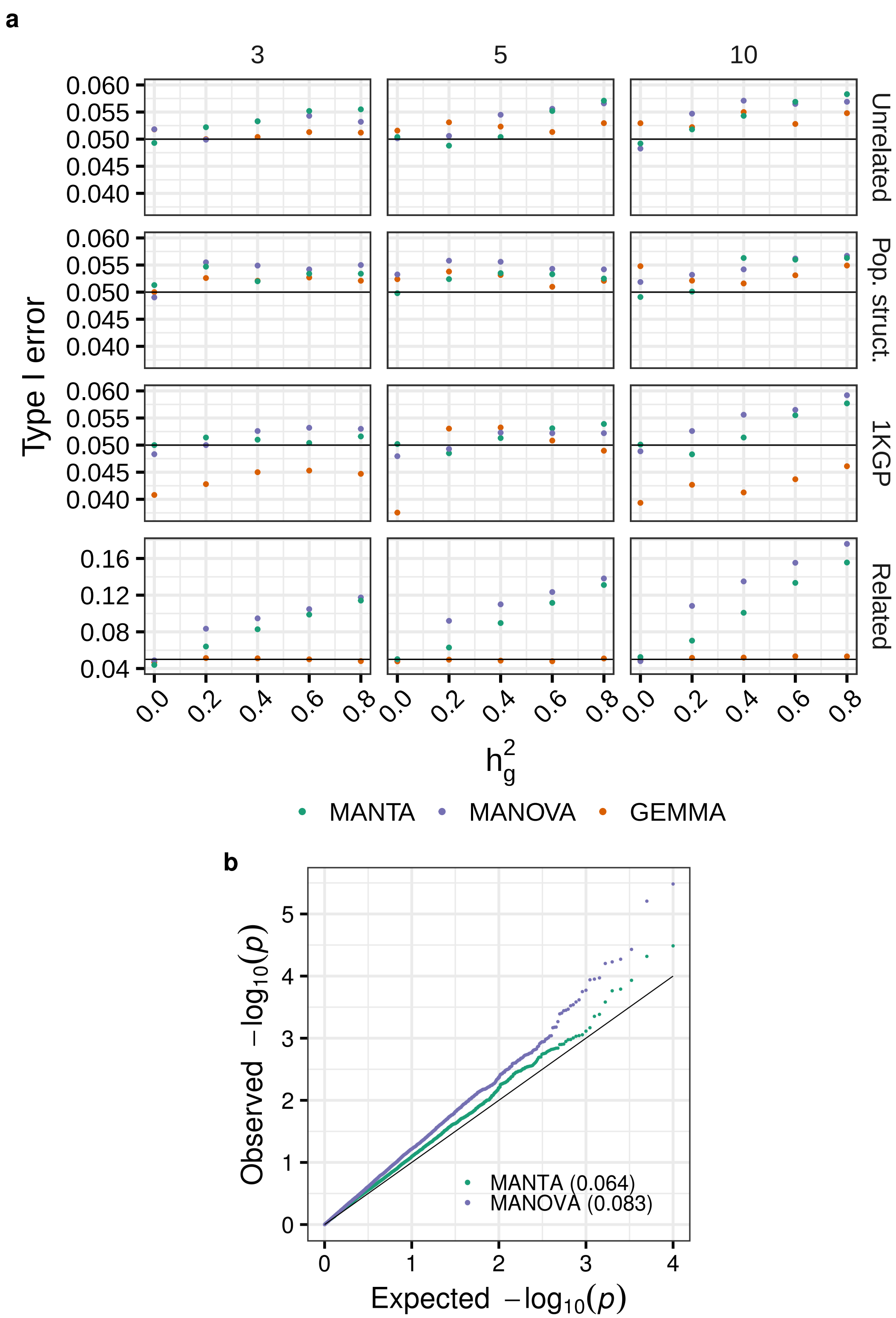

Fig. S8. a) Type I error of MANTA (green), MANOVA (purple) and GEMMA (orange) as a function of the fraction of variance explained by population structure h_g^2, across different numbers of traits (columns) and population structures (rows): unrelated individuals ($n = 1000$), population stratification ($n = 1000$), actual 1000 Genomes Project dataset (1KGP, $n = 2504$) and related individuals ($n = 1000$). Simulation details: multivariate normal residuals, 5 genotype principal components (PCs) included as covariates in the model in the case of MANTA and MANOVA. **b)** QQ-plots of *p* values obtained with MANTA (green) and MANOVA (purple) in a cohort of related individuals, when the fraction of variance explained by population structure is small ($h_g^2 = 0.2$). Type I errors are also shown. Simulation details as above.

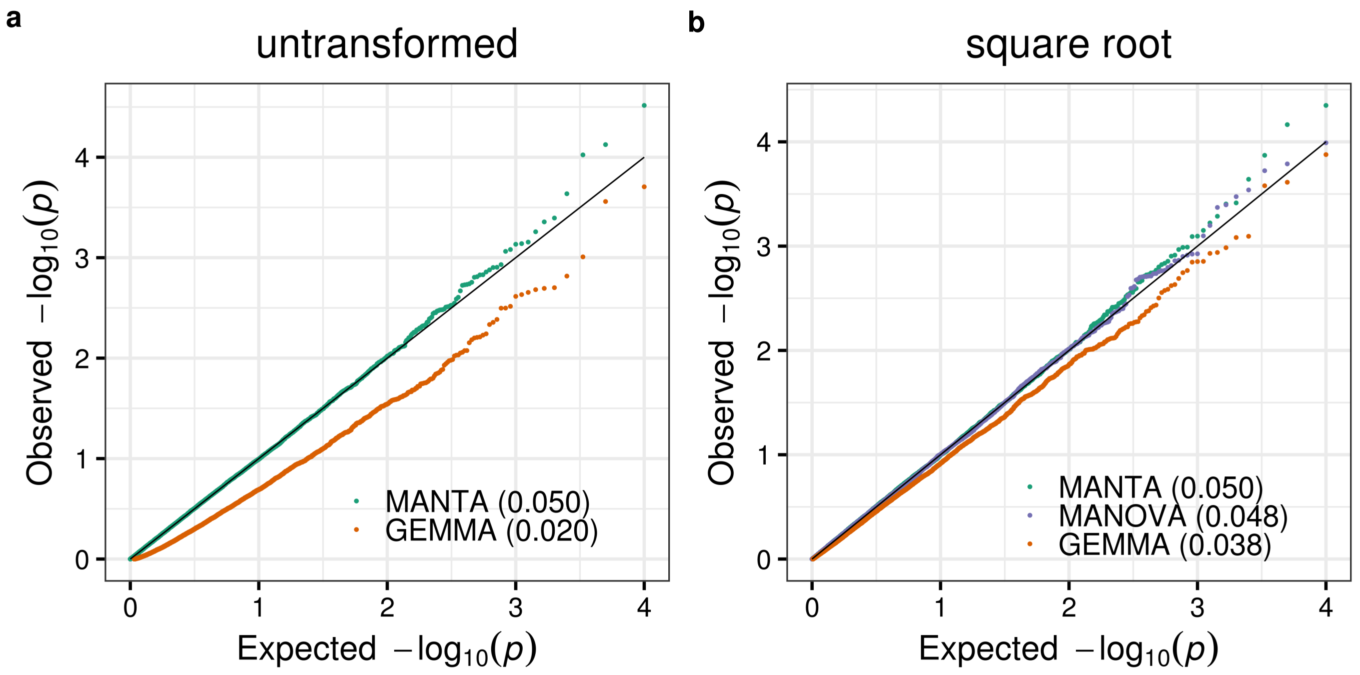

Fig. S9. a) QQ-plots of *p* values obtained with MANTA (green) and GEMMA (orange) when simulating multivariate proportion traits (not residuals, see Methods). Type I errors are also shown. Simulation details: actual 1000 Genomes Project genotypes ($n = 2504$), $q = 5$, 5 genotype principal components (PCs) included as covariates in the model (in the case of MANTA). Note that, in this scenario, MANOVA *p* values cannot be computed. **b)** Analogous representation when the traits are square root transformed. In this case the results of MANOVA are also displayed (purple). Simulation details as above.

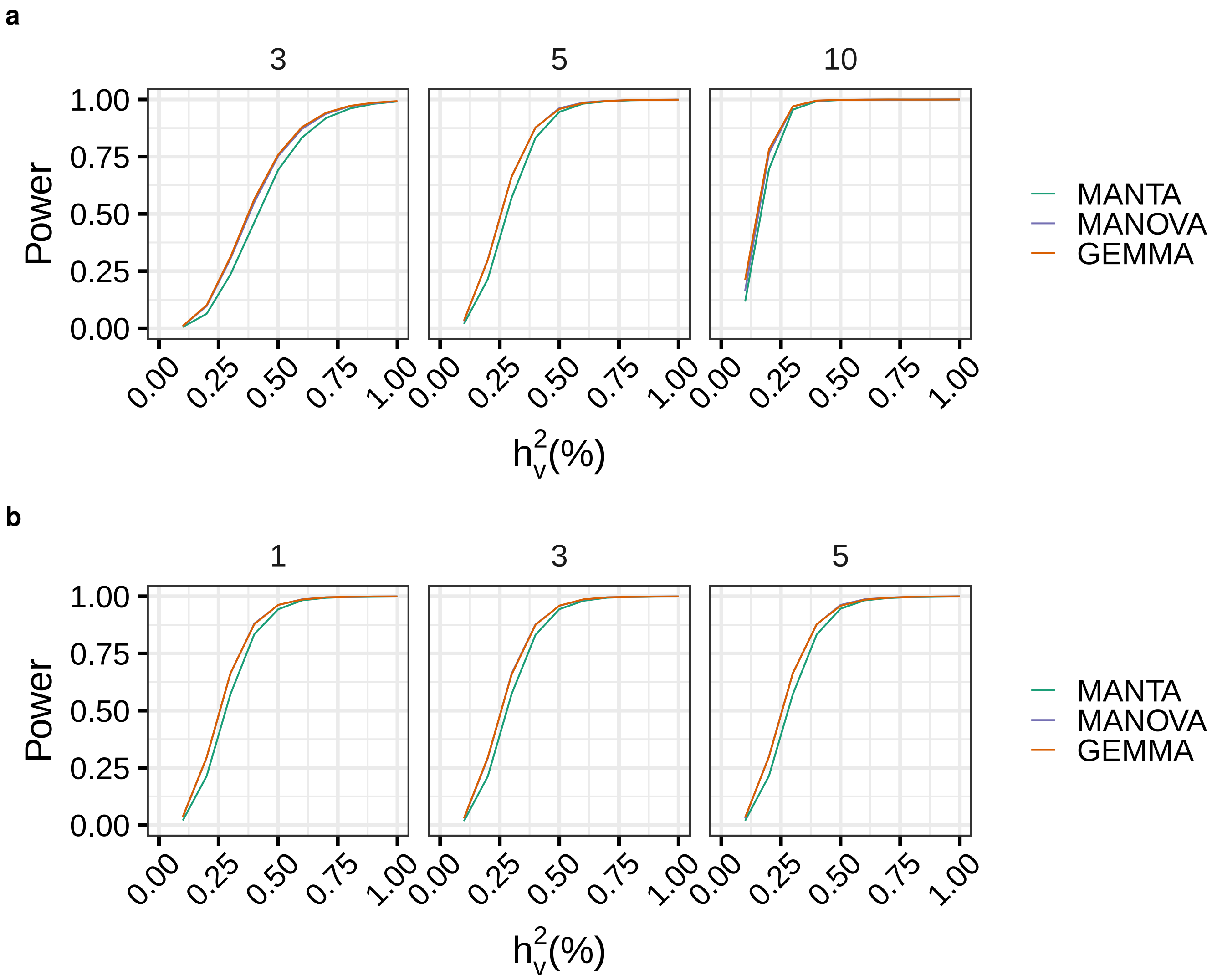

Fig. S10. a) Power of MANTA (green), MANOVA (purple) and GEMMA (orange) as a function of the percentage of variance explained by the causal variant (h_v^2) and the total number of responses (q, all affected by the causal variant, columns) **b)** Analogous representation as a function of the number of affected responses, when this is different from q (here $q = 5$). Simulation details: actual 1000 Genomes Project genotypes ($n =$ 2504), multivariate normal residuals, $h_g^2 =$ 0.2, 5 genotype principal components (PCs) included as covariates in the model in the case of MANTA and MANOVA, Bonferroni corrected *p* values. GEMMA often fails (produces an error) when the number of traits is relatively large: in the $q =$10 scenario shown in a), on average, one out of two GEMMA tests resulted in an error. The fraction of errors in scenarios with fewer traits was substantially smaller (see Methods).

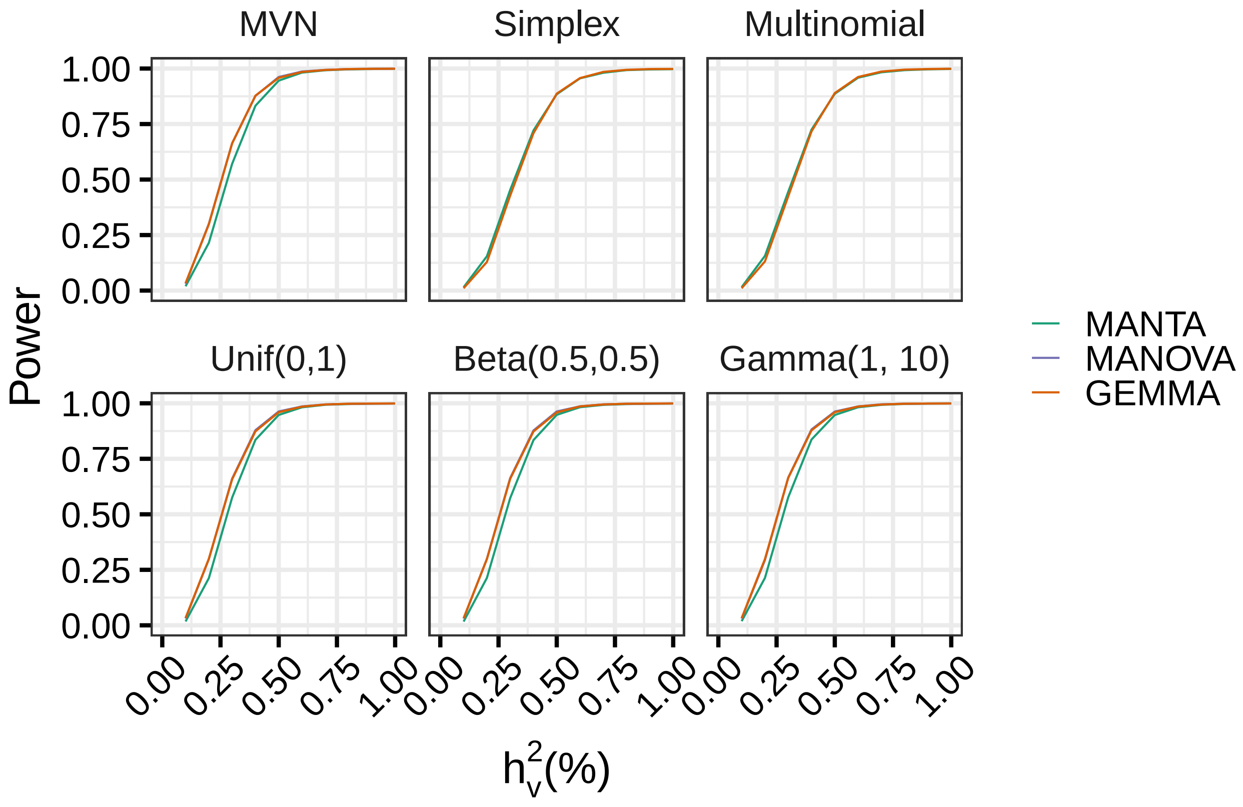

Fig. S11. Power of MANTA (green), MANOVA (purple) and GEMMA (orange) as a function of the percentage of variance explained by the causal variant (h_v^2) and the residual distribution. Simulation details: actual 1000 Genomes Project genotypes ($n = 2504$), $q = 5$ traits, $h_g^2 = 0.2$, 5 genotype principal components (PCs) included as covariates in the model in the case of MANTA and MANOVA, Bonferroni corrected *p* values. Note that MANOVA *p* values cannot be calculated for linearly dependent traits, as it is the case of the simplex and multinomial scenarios.

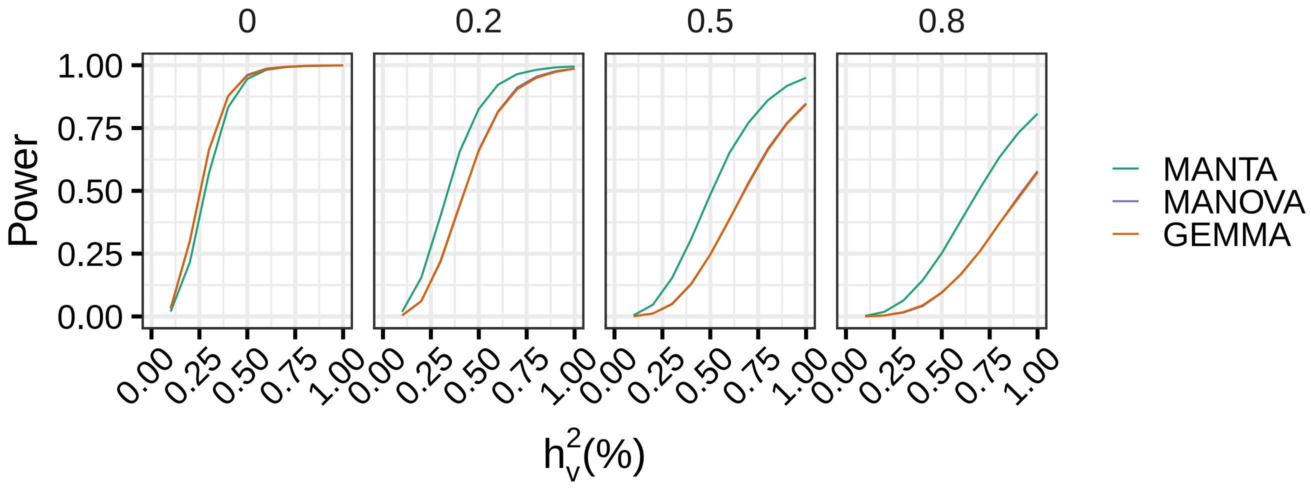

Fig. S12. Power of MANTA (green), MANOVA (purple) and GEMMA (orange) as a function of the percentage of variance explained by the causal variant (h_v^2), for increasing values of trait-to-trait correlations. Simulation details: actual 1000 Genomes Project genotypes ($n = 2504$), $q = 5$ traits, multivariate normal residuals, $h_g^2 = 0.2$, 5 genotype principal components (PCs) included as covariates in the model in the case of MANTA and MANOVA, Bonferroni corrected *p* values.

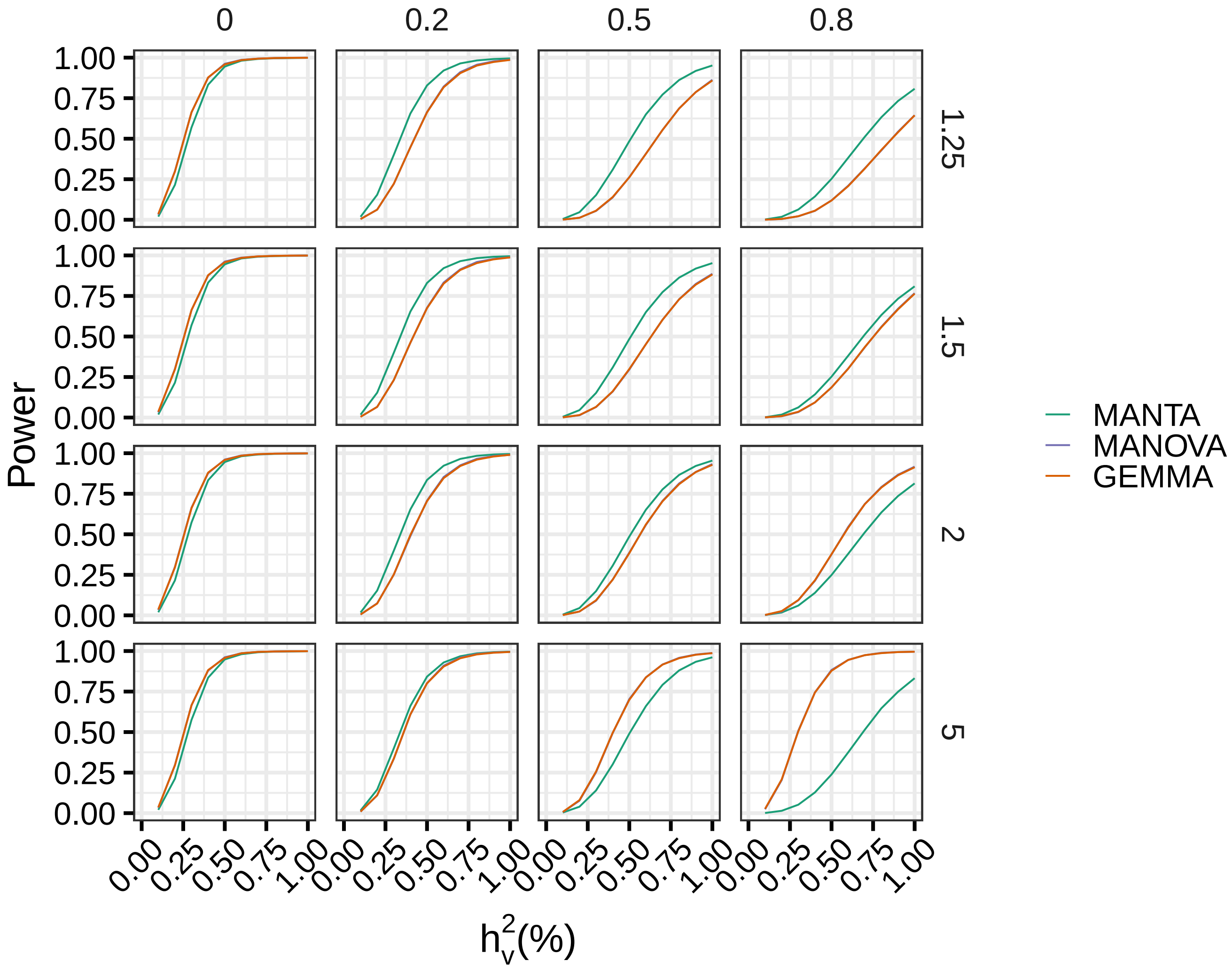

Fig. S13. Power of MANTA (green), MANOVA (purple) and GEMMA (orange) as a function of the percentage of variance explained by the causal variant (h_v^2), across trait-to-trait correlations (columns) and different effect size max/min ratios (rows). A max/min effect size ratio of 1 corresponds to simulate equal (unit) effect sizes across all the traits, that is, effects are concordant with the trait correlation structure. As the max/min effect size ratio increases, the more discordant are genetic effects and trait correlations (see Methods). Simulation details: actual 1000 Genomes Project genotypes ($n = 2504$), $q = 5$ traits, multivariate normal residuals, $h_g^2 = 0.2$, 5 genotype principal components (PCs) included as covariates in the model in the case of MANTA and MANOVA, Bonferroni corrected *p* values.

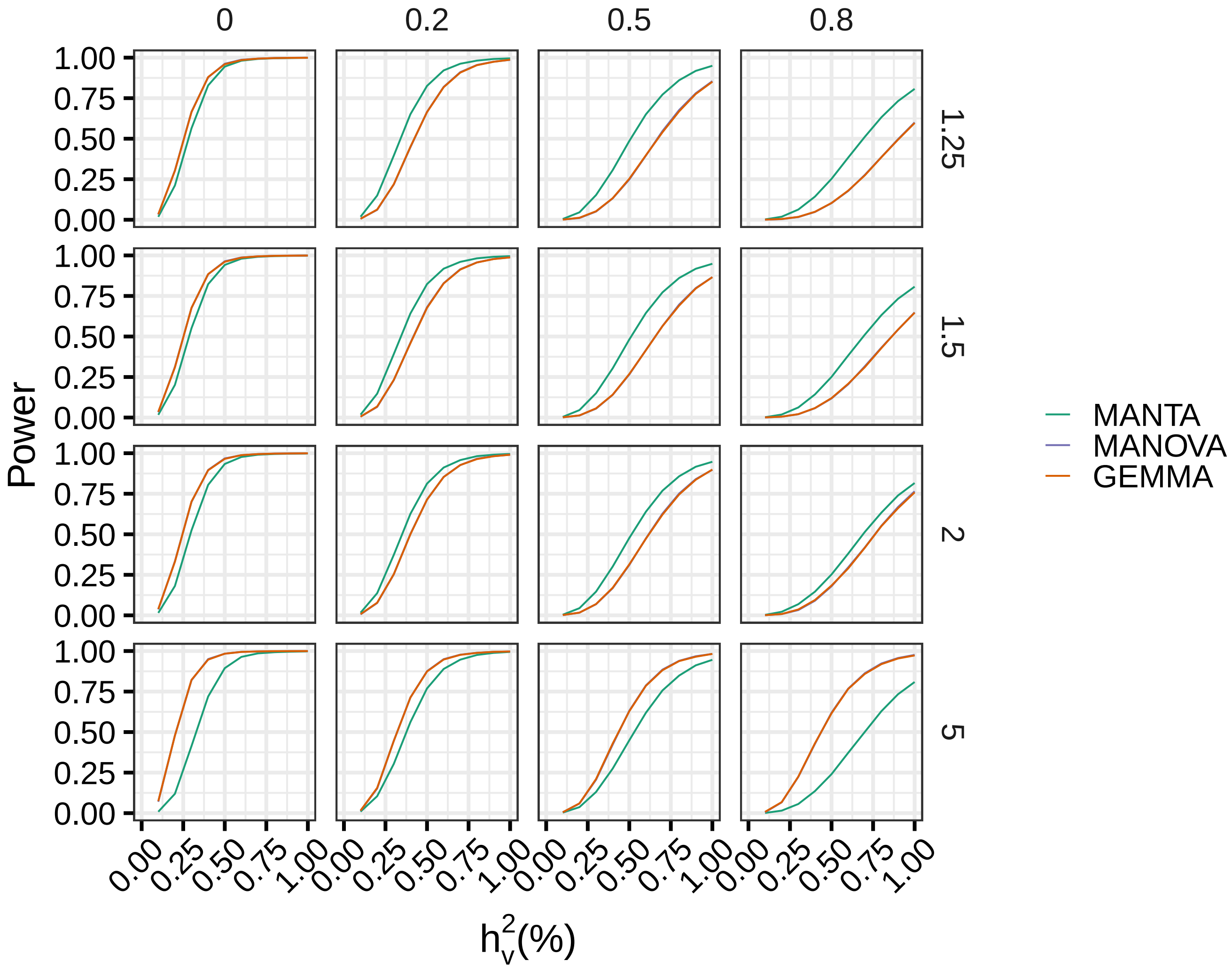

Fig. S14. Power of MANTA (green), MANOVA (purple) and GEMMA (orange) as a function of the percentage of variance explained by the causal variant (h_v^2), across trait-to-trait correlations (columns) and trait max/min variance ratios (rows). A max/min variance ratio of 1 corresponds to equal (unit) variances simulated for all traits (see Methods). Simulation details: actual 1000 Genomes Project genotypes (n = 2504), q = 5 traits, multivariate normal residuals, h_g^2 = 0.2, 5 genotype principal components (PCs) included as covariates in the model in the case of MANTA and MANOVA, Bonferroni corrected *p* values.

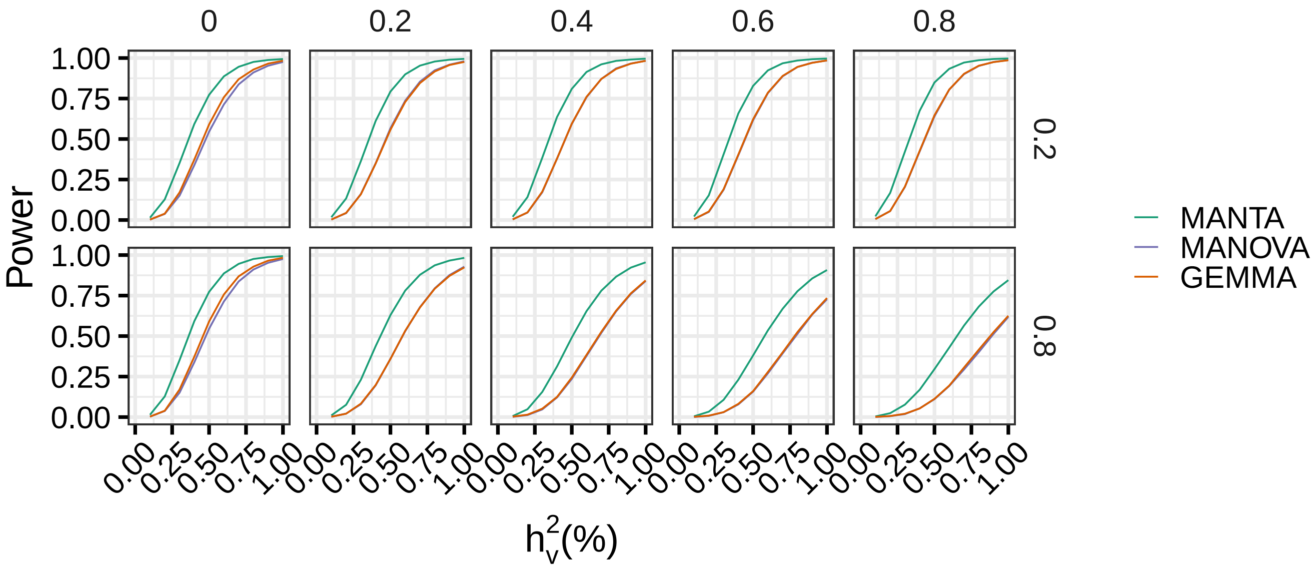

Fig. S15. Power of MANTA (green), MANOVA (purple) and GEMMA (orange) as a function of the percentage of variance explained by the causal variant (h_v^2), for different values of the fraction of variance explained by population structure, h_g^2 (columns), and trait-to-trait correlations due to population structure (rows). Simulation details: actual 1000 Genomes Project genotypes ($n = 2504$), $q = 5$ traits, multivariate normal residuals, 5 genotype principal components (PCs) included as covariates in the model in the case of MANTA and MANOVA, Bonferroni corrected *p* values.

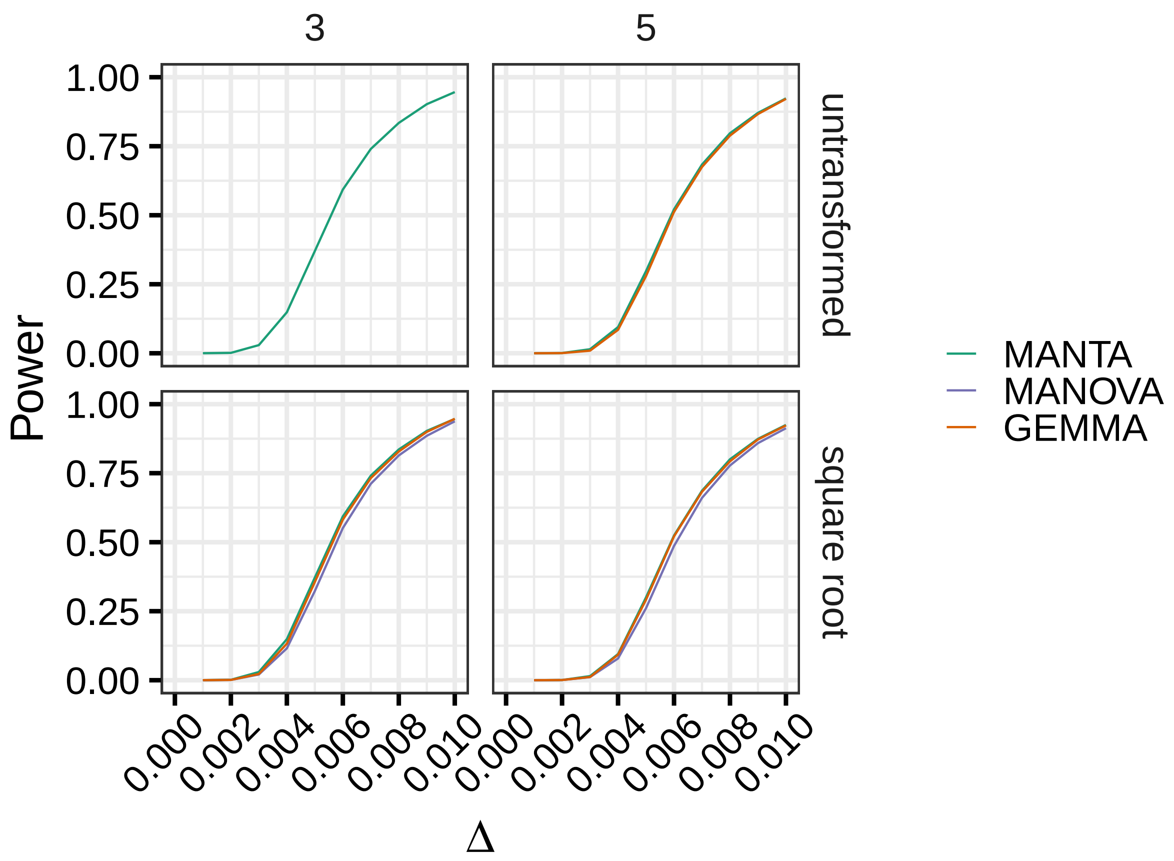

Fig. S16. Power of MANTA (green), MANOVA (purple) and GEMMA (orange) as a function of Δ, when simulating multivariate proportion traits (not residuals, see Methods), for different number of responses (columns) and trait transformations (rows). Simulation details: actual 1000 Genomes Project genotypes ($n = 2504$), 5 genotype principal components (PCs) included as covariates in the model (in the case of MANTA), Bonferroni corrected *p* values. Note that MANOVA *p* values cannot be computed and that GEMMA often fails (produces an error and does not generate any result) with untransformed traits in this scenario (see Methods).

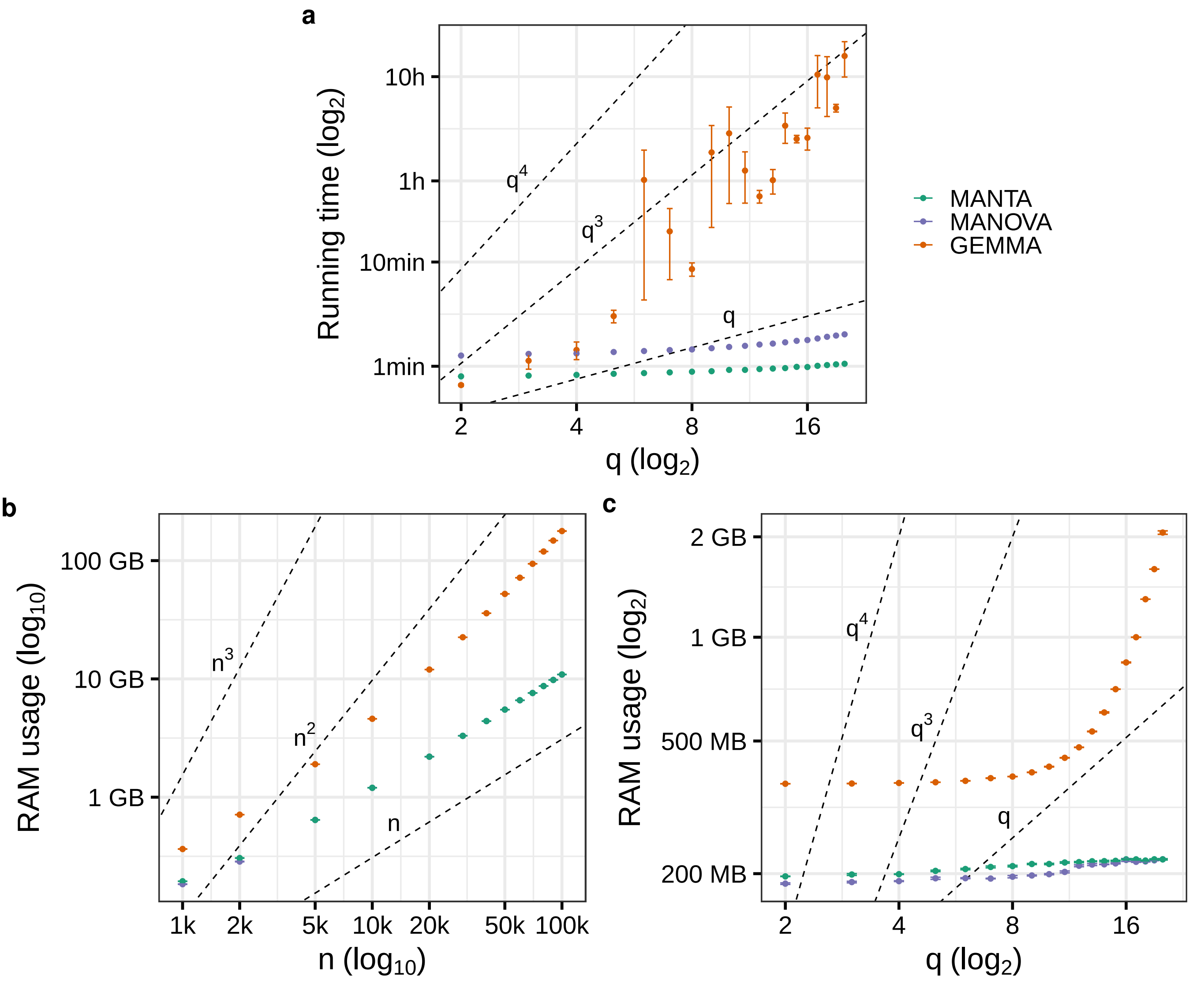

Fig. S17. a) Empirical running time of MANTA (green), MANOVA (purple) and GEMMA (orange) as a function of the number of traits (q). Each point corresponds to the mean running time across 5 runs with different input data (see Methods). Error bars represent the standard error of the mean (i.e. mean $\pm$ SEM). Axes are in $\log_2$ scale. Dashed lines represent running time growth rates of q, q^3 and q^4. **b)**, **c)** Analogous representations for the RAM usage (peak resident set size) of the three methods as a function of the sample size (n) and the number of traits (q), respectively.

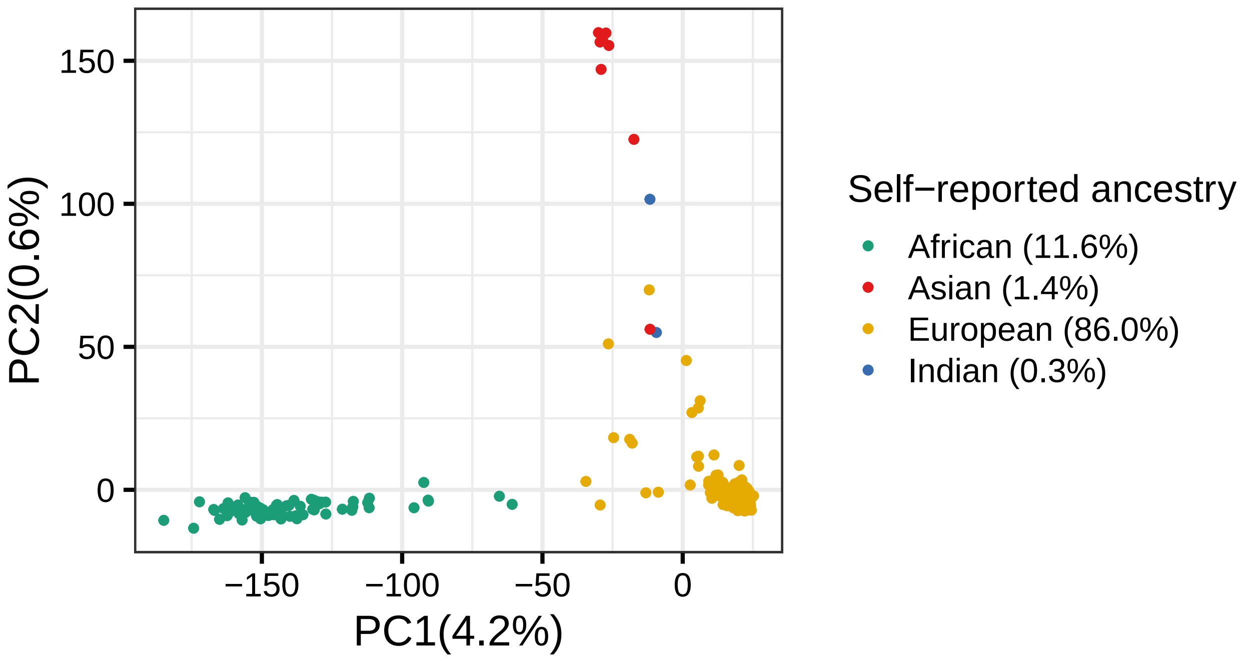

Fig. S18. Principal Component Analysis (PCA) based on the genotypes of GTEx individuals. Colors represent self-reported ancestry (by the donor, family/next of kin or medical record). Individuals of unknown ancestry (0.6%) are not shown. The percentage of variance explained by the two first PCs and the percentage of individuals of each ancestry appear between parentheses. Self-reported ancestry matches the patterns observed in the PCA. Only individuals of European and African ancestry were used for pb-sQTL mapping.

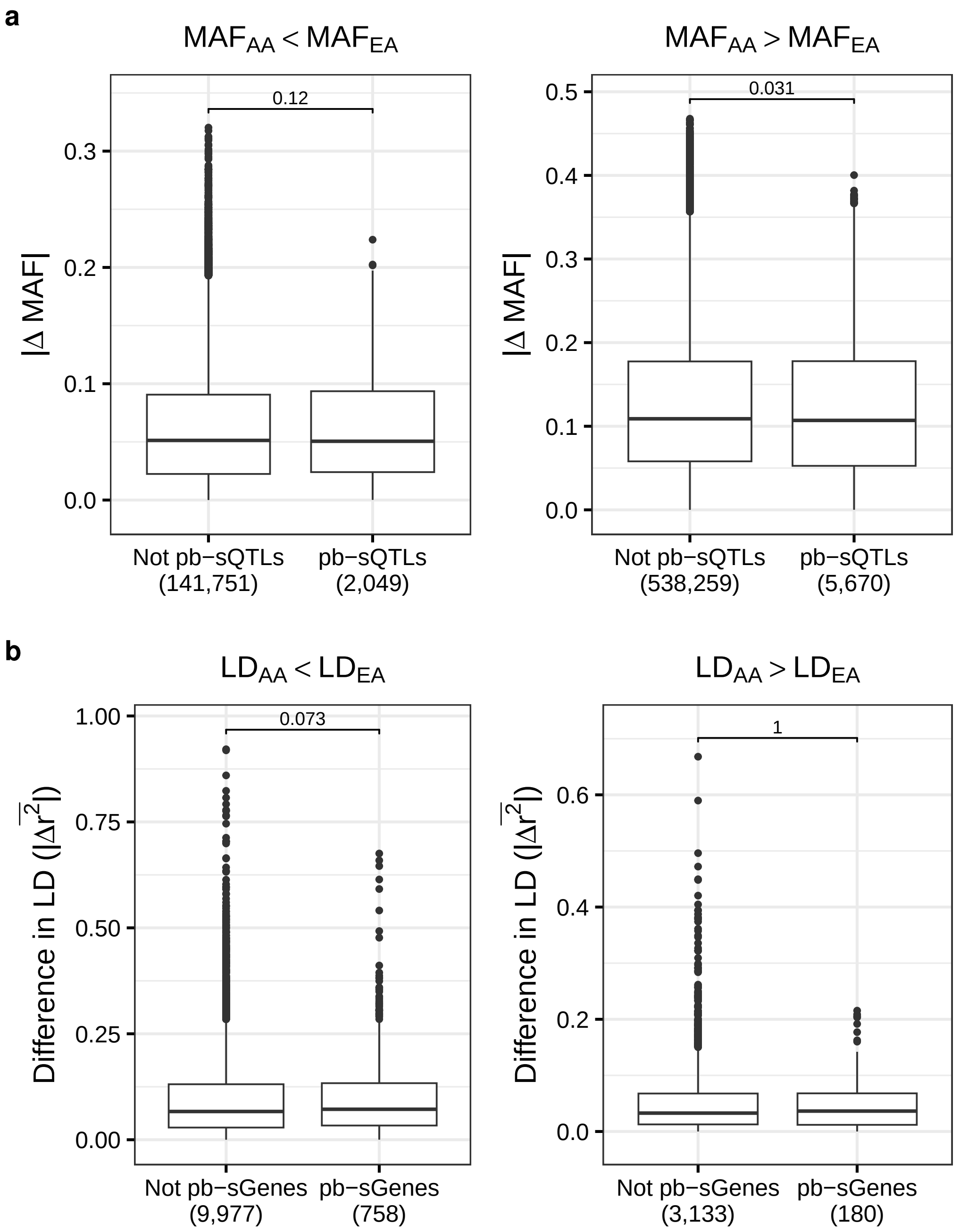

Fig. S19. a Distribution of the absolute differences in minimum allele frequency (MAF) between EA and AA individuals, for pb-sQTLs and not pb-sQTLs. The number of variants is shown between parenthesis. Wilcoxon Rank-Sum test *p* values are also displayed. **b** Distribution of the absolute differences in linkage disequilibrium (LD) between EA and AA individuals for pb-sGenes and not pb-sGenes. LD was estimated as the average r^2 between all pairs of variants tested for association with a given gene. The number of genes is shown between parentheses. Wilcoxon Rank-Sum test *p* values are also displayed. In boxplots, the box represents the first to third quartiles and the median, while the whiskers indicate $\pm$ 1.5 $\times$ interquartile range.

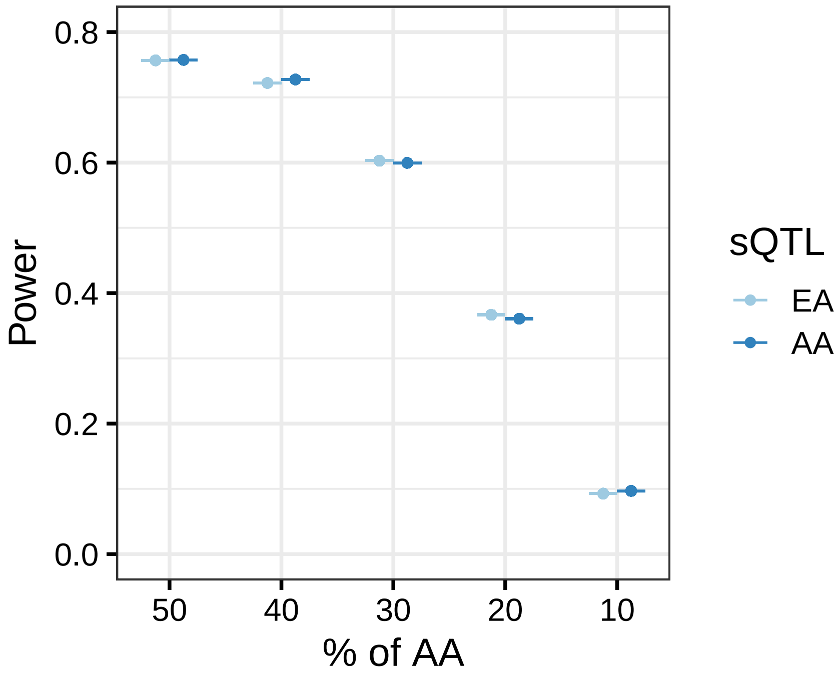

Fig. S20. Power to detect EA-specific (light blue) and AA-specific (dark blue) sQTLs as a function of sample size imbalance, measured by the percentage of AA individuals (x-axis). Points correspond to the mean power across 100 simulation runs. Error bars represent the standard error of the mean (i.e. mean ± SEM).

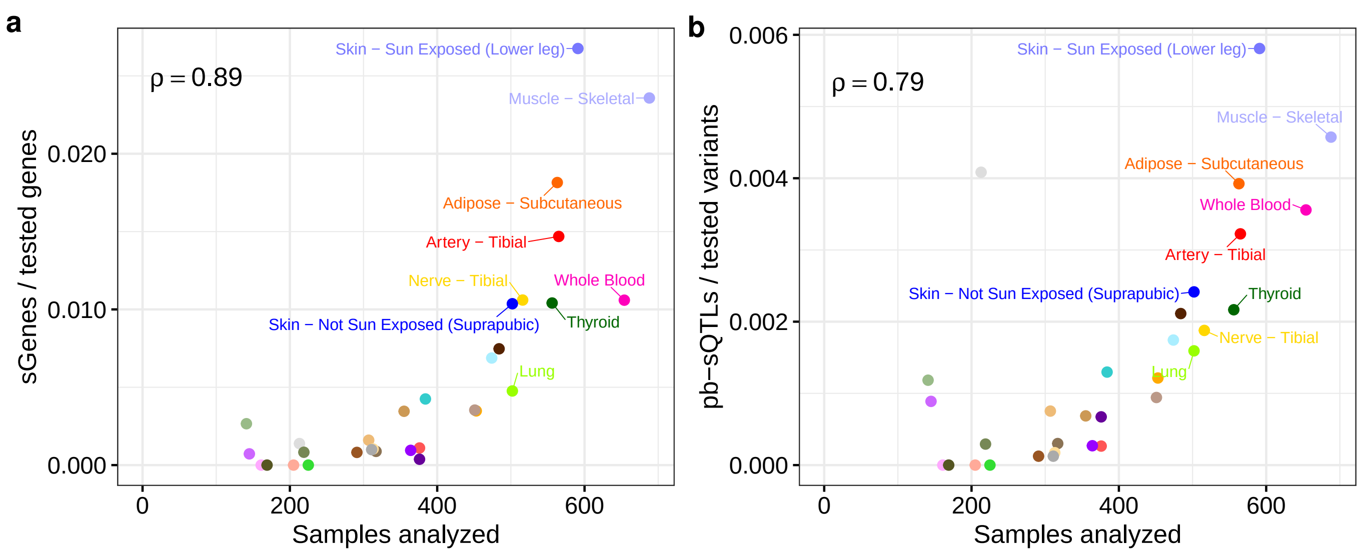

Fig. S21. **a)**. Proportion of genes with pb-sQTLs (over tested genes, y-axis) per tissue with respect to the tissue sample size (x-axis). Spearman correlation between both metrics is also displayed. Tissues with sample size $>$ 500 are labelled. Tissue color codes are available in Additional file 1: Table S1. **b)**. Analogous representation for the proportion of tested variants identified as pb-sQTLs.

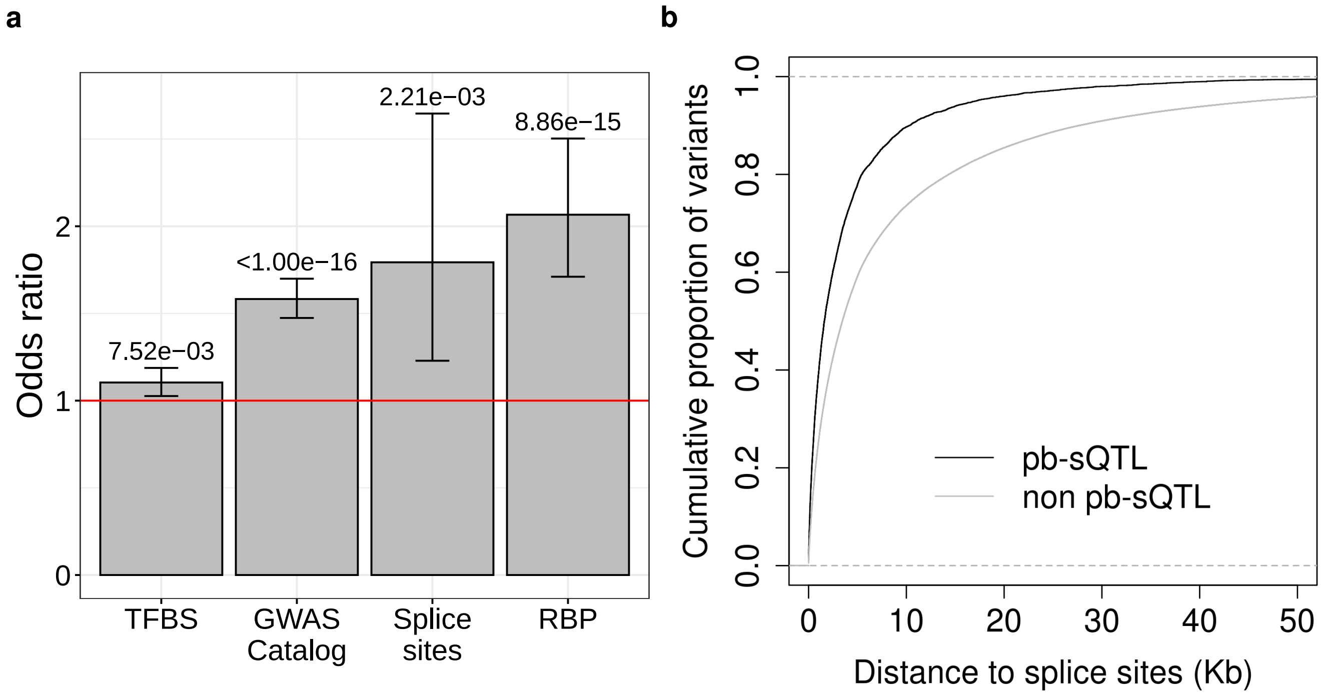

Fig. S22. a) Enrichment of pb-sQTLs, with respect to a set of matched non pb-sQTLs (two-sided Fisher's exact test FDR < 0.05), in a set of functional categories: transcription factor binding sites (TFBS) from the Ensembl Regulatory Build, GWAS Catalog hits, splice sites from protein-coding and lincRNA genes annotated in GENCODE v26 and ENCODE RNA-binding protein (RBP) eCLIP peaks (see Methods). For each functional category, the height of the bar represents the enrichment odds ratio (OR), and the error bar its 95% confidence interval. FDR values are also displayed. **b)** Cumulative distribution of the distance to the closest splice donor or acceptor splice site from genes protein-coding genes and lincRNAs annotated in GENCODE v26, both for pb-sQTLs and non pb-sQTLs.

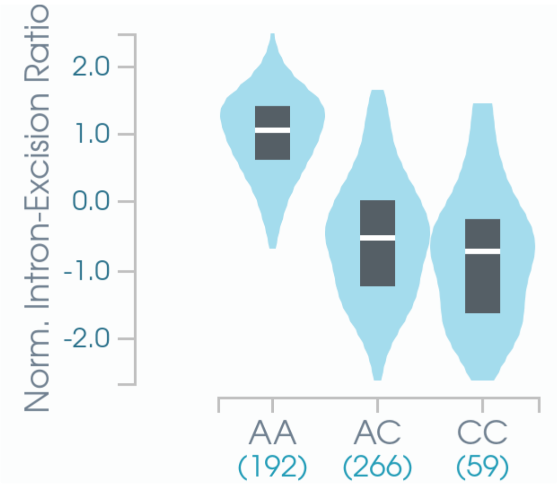

Fig. S23. Normalized Leafcutter's intron-excision ratio (y-axis) for intron chr19:50,952,668-50,952,747 of *KLK5*, in individuals with different genotype at rs2739412 (chr19:50,952,558, A/C). The number of individuals with each genotype is shown between parentheses. This plot was directly obtained from the GTEx Portal (`https://gtexportal.org`).

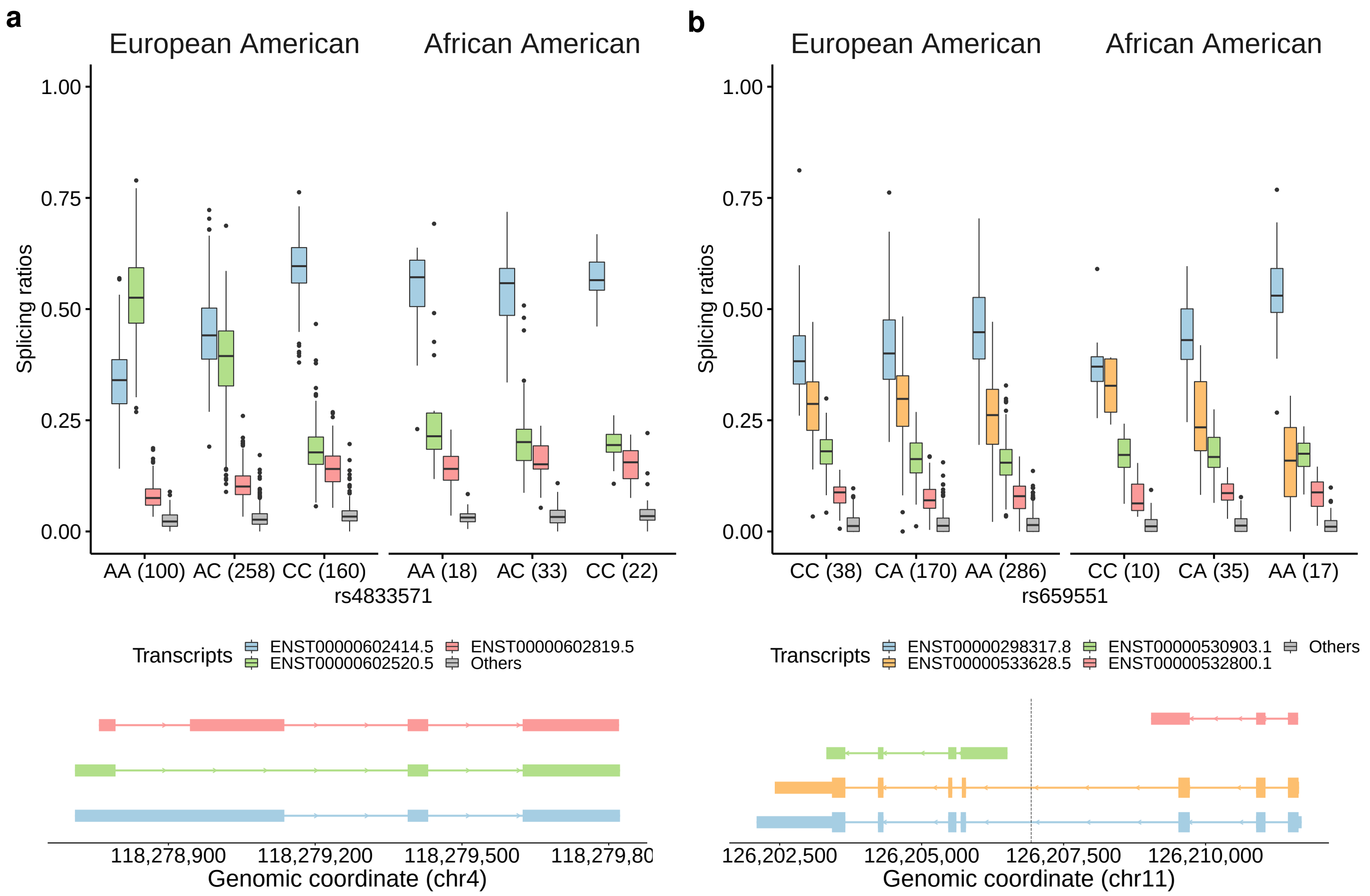

Fig. S24. **a)** Relative abundances of the most expressed isoforms in skin (sun-exposed) from the lincRNA *SNHG8* (chr4:118,278,709-118,285,316, forward strand), for each ancestry (European American, EA, African American, AA) and genotype group at the rs4833571 locus (chr4:118,283,565 A/C), shown as boxplots. In boxplots, the box represents the first to third quartiles and the median, while the whiskers indicate $\pm$ 1.5 $\times$ interquartile range. The least abundant isoforms are grouped in Others. The number of individuals in each genotype group is shown between parentheses. In European Americans, reference homozygous (AA) individuals express preferentially the *ENST00000602520.5* (green) isoform, while in alternative homozygous (CC) individuals, isoform *ENST00000602414.5* (blue) captures most of the expression of the gene. Heterozygous EA individuals (TC) display an intermediate behavior. In African Americans, however, isoform expression does not change with the genotype at rs4833571, with *ENST00000602414.5* (blue) being the most expressed isoform in all cases. The exonic structure of the most expressed isoforms of *SNHG8* in skin (sun-exposed) is also displayed. rs4833571 is an pb-sQTL for *SNHG8* in 6 additional tissues. **b)** Analogous representation for rs659551 (chr11:126,206,929, C/A), a pb-sQTL for gene *RPUSD4* (chr11:126,202,096-126,211,692, reverse strand, protein-coding) in thyroid. In this case, in African Americans, the abundance of isoform *ENST00000298317.8* (blue) increases, while the abundance of *ENST00000533628.5* (orange) decreases, with the number of alternative alleles (A) at the rs659551 locus. This behavior can be barely observed in European Americans. *RPUSD4* is a thyroid-specific pb-sGene. The exonic structure of the most expressed isoforms of *RPUSD4* in thyroid is also shown, together with the location of the SNP (dotted vertical line).

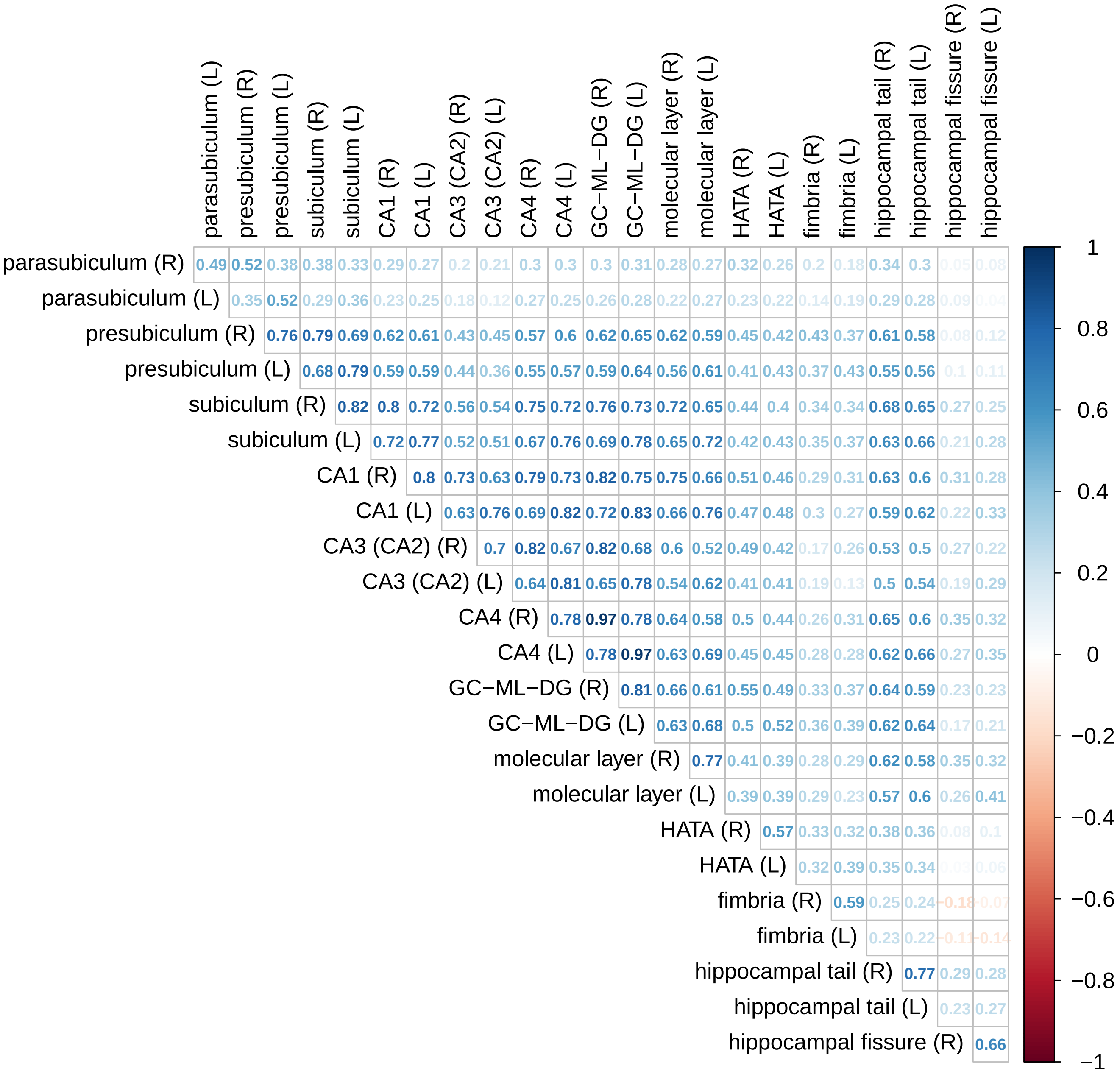

Fig. S25. Pairwise Pearson correlation of the volume of hippocampal subfields in right (R) and left (L) brain hemispheres.

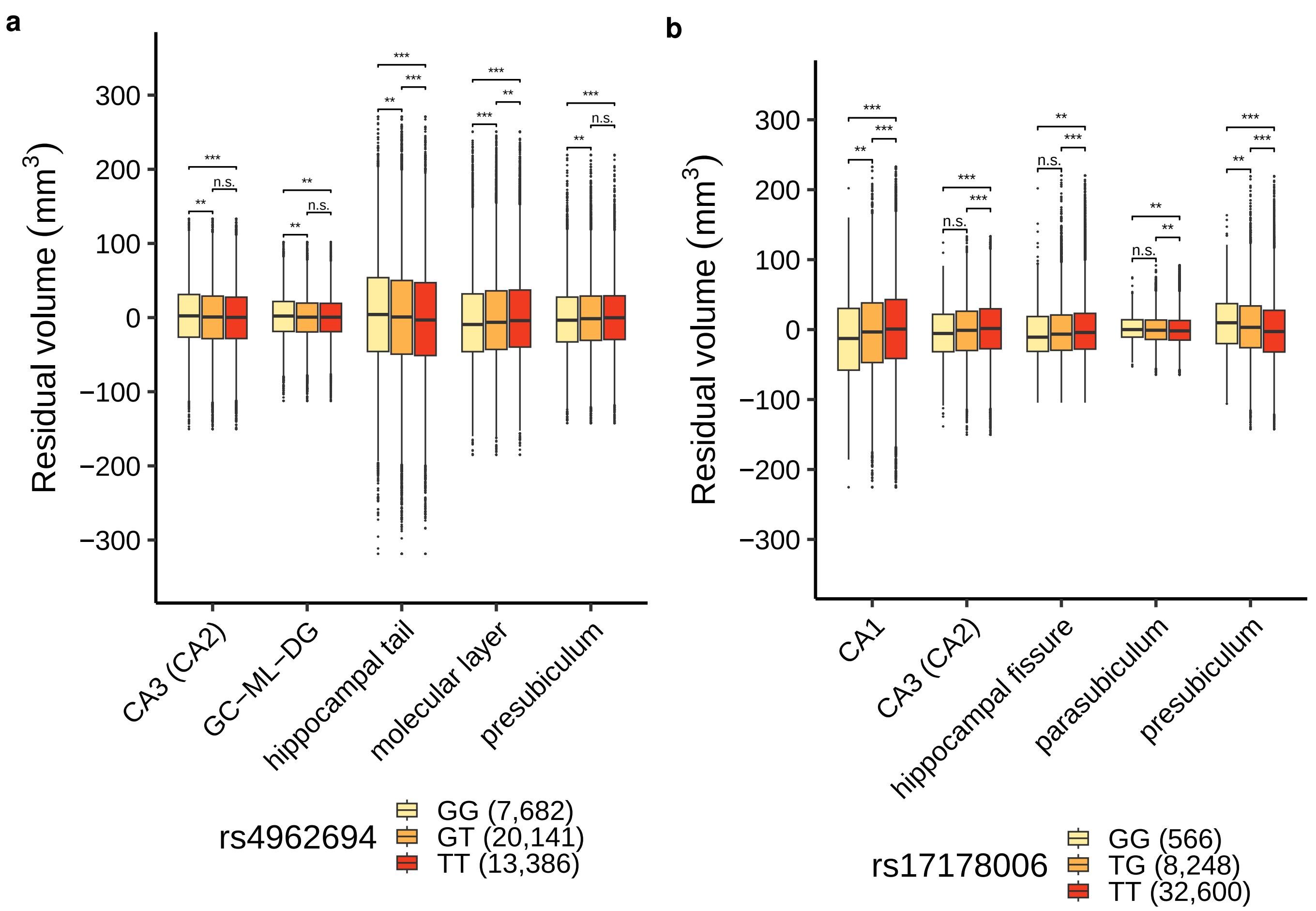

Fig. S26. a) Covariate-adjusted volumes (mm^3) of the most changing hippocampal subfields across genotype groups at rs4962694 (chr10:126,436,717, G/T) are shown as boxplots. In boxplots, the box represents the first to third quartiles and the median, while the whiskers indicate $\pm$ 1.5 $\times$ interquartile range. To improve visualization, 99.9% winsorization was applied. The number of individuals on each group is shown between parentheses. FDR adjusted *p* values (Wilcoxon Rank-Sum test) for each pairwise comparison are also displayed, encoded as follows: *** ($p \leq$ 0.001), ** ($0.001 < p \leq 0.01$), * ($0.01 < p \leq 0.05$), n.s. ($p > 0.05$). rs4962694 was previously associated with the volume of the molecular layer, but not with the volume of other subfields[4]. **b)** Analogous representation for rs17178006 (chr12:65,718,299, T/G). This SNP was previously associated with the volume of presubiculum and CA1 (although associations with the volumes of CA3 (CA2), CA4, granule cell layer of the DG and hippocampal fissure were also reported when not correcting for total hippocampal volume)[4].

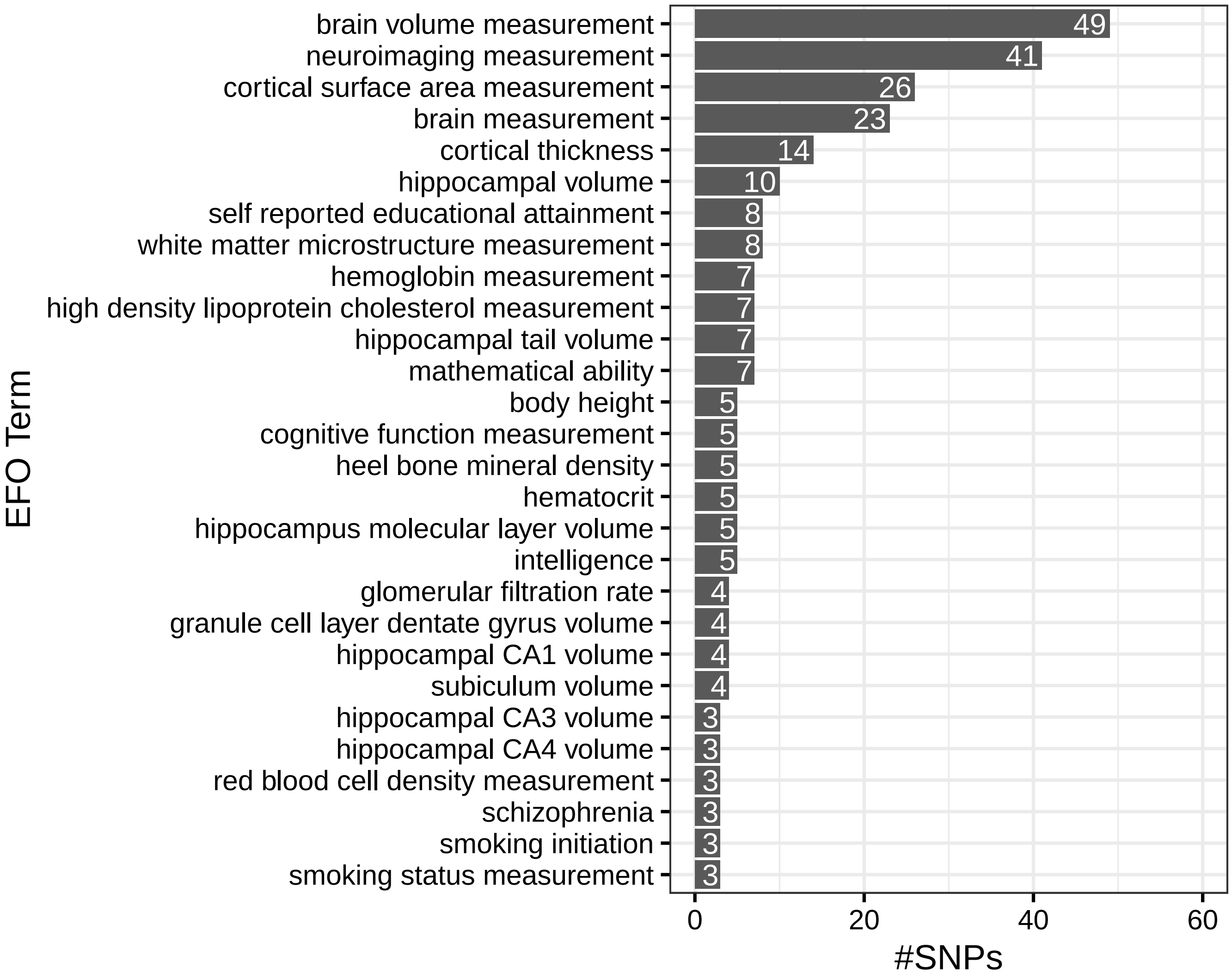

Fig. S27. Top Experimental Factor Ontology (EFO) terms corresponding to the GWAS traits associated with the genetic variants identified by our approach. The number of genetic variants associated with each EFO term is also displayed. Note that the same genetic variant can be associated with more than one EFO term. Information retrieved from the GWAS Catalog (accessed 2021-01-29).

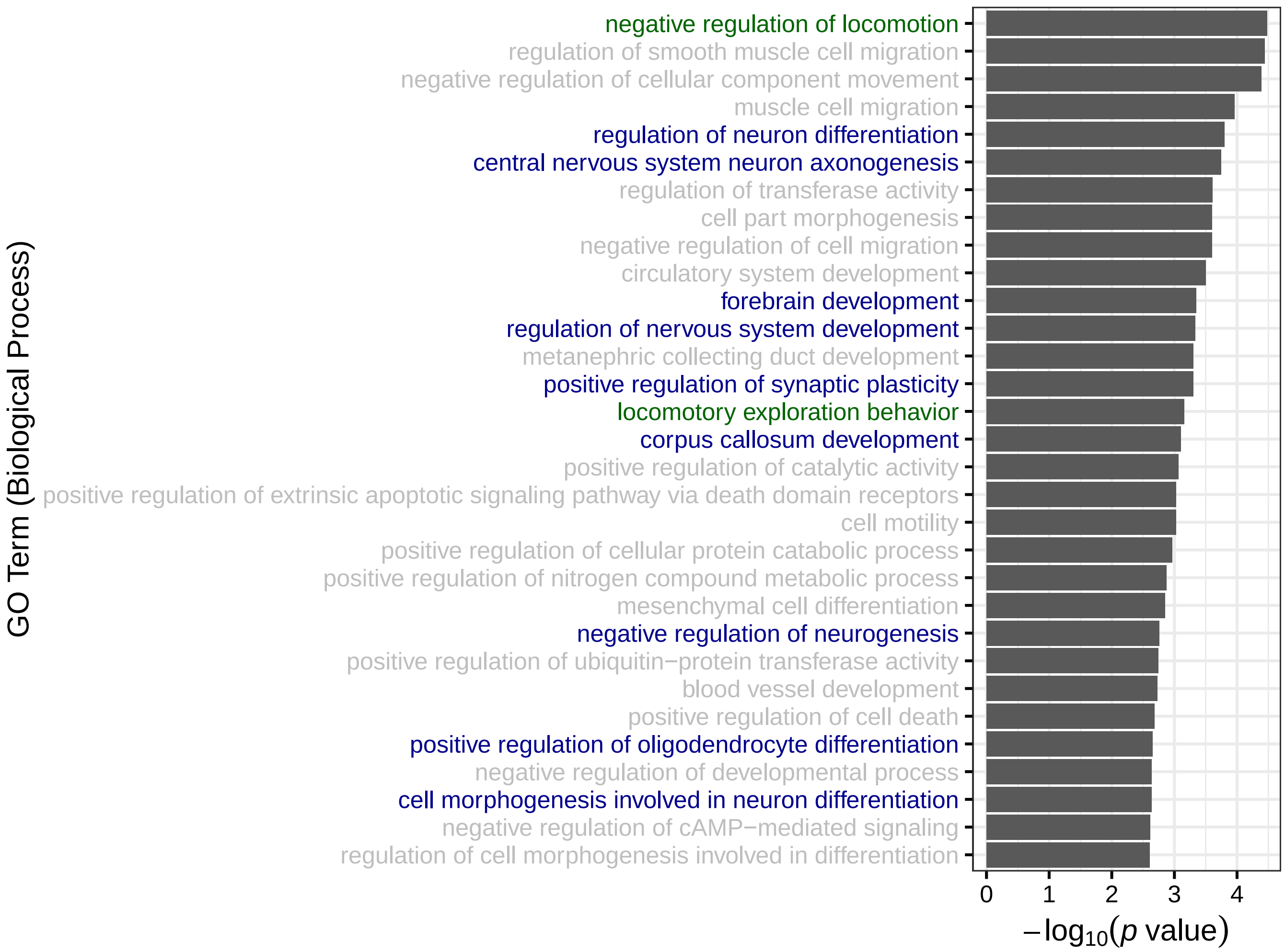

Fig. S28. Gene ontology (GO) Biological Process terms enriched among the closest genes to the genetic variants associated with hippocampal subfield volumes (FDR < 0.1). For each term (y-axis), the corresponding $-\log_{10}$ p value (hypergeometric test) is shown (x-axis). Terms related to neuronal development and differentiation (blue), as well as to locomotive and exploratory behavior (green) are highlighted.

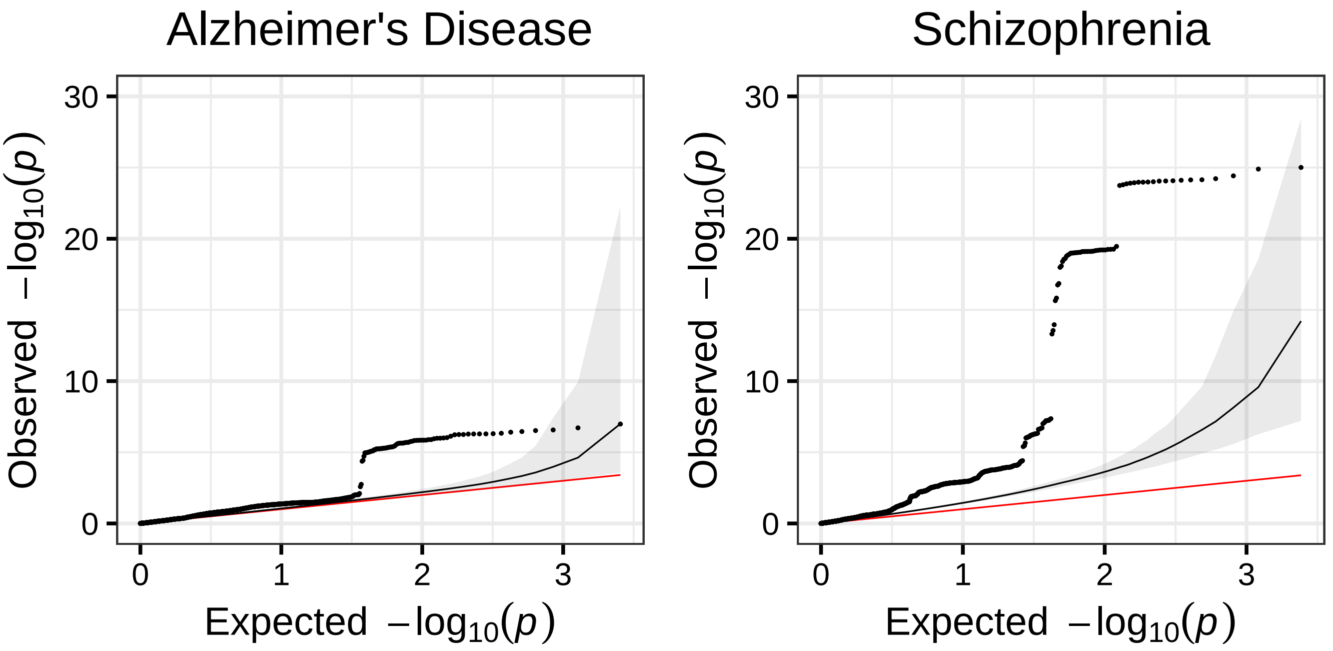

Fig. S29. Quantile-quantile (QQ) plots of *p* values for association with Alzheimer's disease (left) and schizophrenia (right), for genetic variants associated with hippocampal subfield volumes (asymptotic PERMANOVA test *p* value $< 5 \cdot 10^{-8}$) plus variants in high linkage disequilibrium with them ($r^2 > 0.8$) (black dots), and the rest of variants (black line and gray area). The black line and gray area represent, respectively, the median and middle 95% observed $-\log_{10}$ *p* values across 10,000 random samplings from the latter variant set, with the same size as the former. The identity line is shown in red.

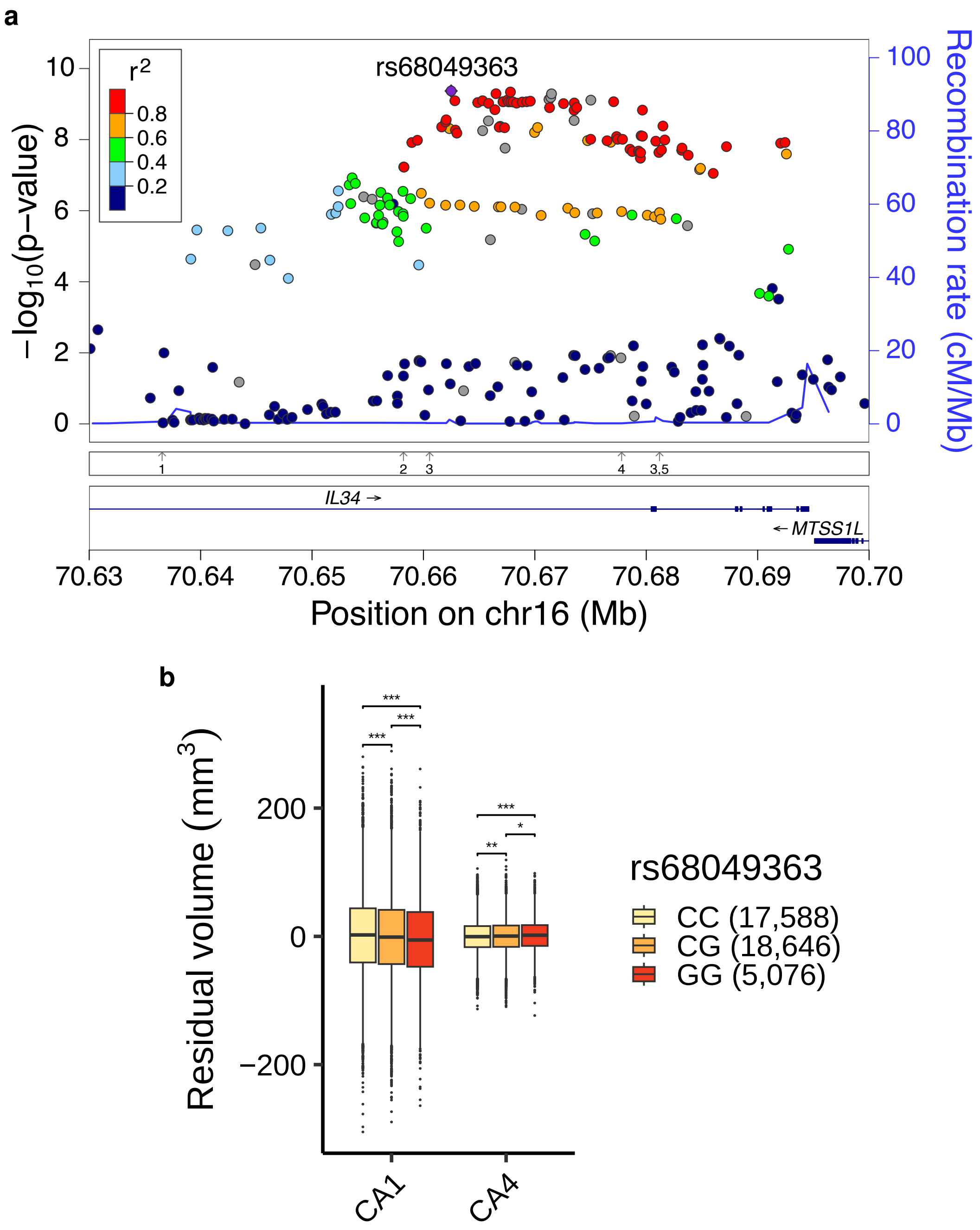

Fig. S30. a) Regional plot of the *IL34* locus, showing the $-\log_{10}$ *p* values for association with hippocampal subfield volumes for all tested genetic variants (asymptotic PERMANOVA test). Linkage disequilibrium patterns (color-coded) and recombination rates are also displayed. The lower panel represents the location of previous associations with brain-related traits in the GWAS Catalog (shown as arrows), encoded as follows: 1) Hippocampal volume in Alzheimer's disease dementia, 2) Cerebrospinal P-tau181p levels, 3) Brain morphology, 4) Cortical surface area, 5) Subcortical volume. **b** Covariate-adjusted volumes (mm^3) of the most changing hippocampal subfields across genotype groups at rs68049363 (chr16:70,662,480, C/G) are shown as boxplots. In boxplots, the box represents the first to third quartiles and the median, while the whiskers indicate $\pm$ 1.5 $\times$ interquartile range. The number of individuals on each group is shown between parentheses. FDR adjusted *p* values (Wilcoxon Rank-Sum test) for each pairwise comparison are also displayed, encoded as follows: *** ($p \leq 0.001$), ** ($0.001 < p \leq 0.01$), * ($0.01 < p \leq 0.05$), n.s. ($p > 0.05$).

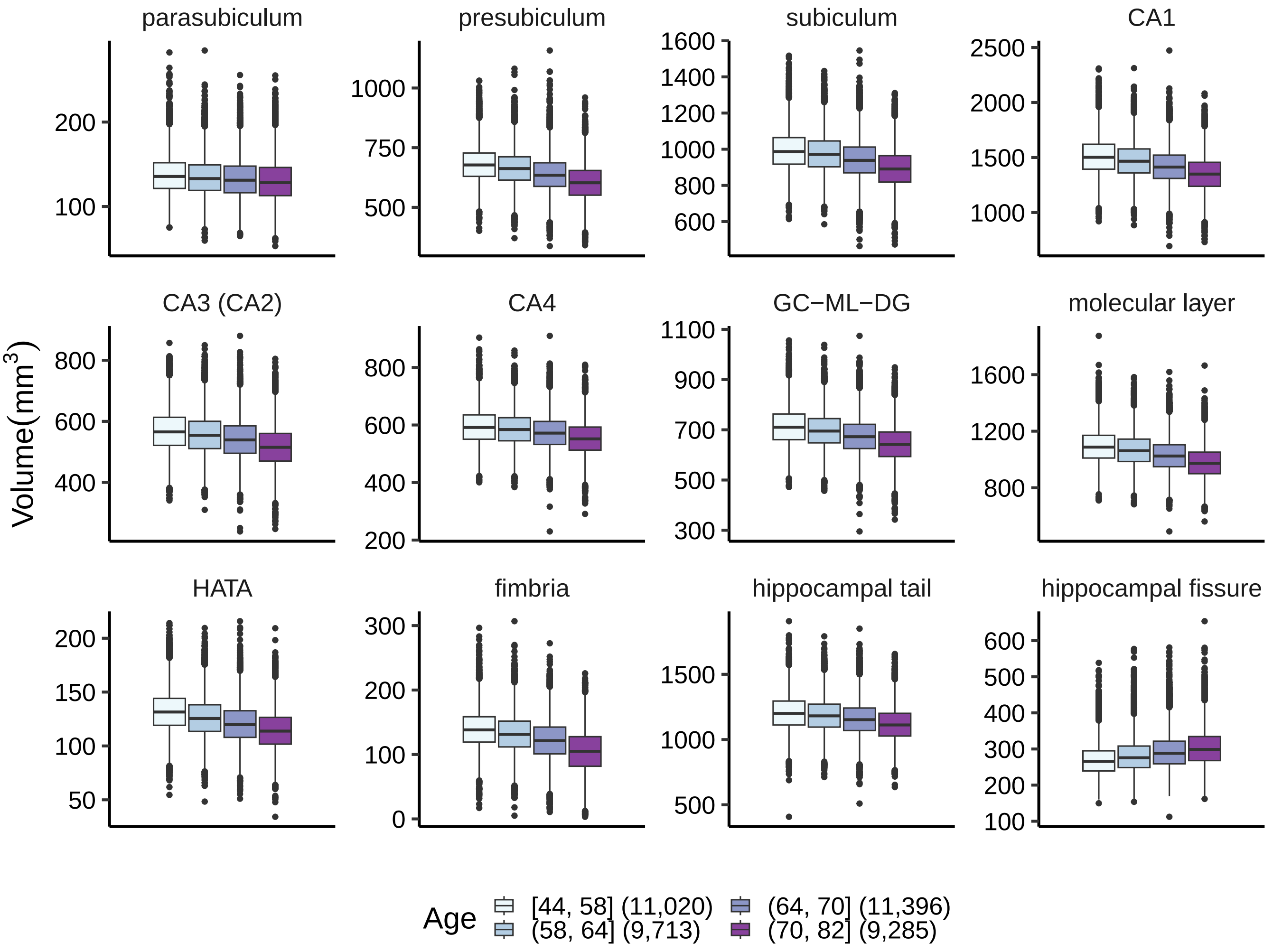

Fig. S31. Distribution of the volumes (mm^3) of 12 hippocampal subfields, represented as boxplots, across four age ranges (corresponding to the quartiles of the age distribution). The number of individuals on each range is shown between parentheses. In boxplots, the box represents the first to third quartiles and the median, while the whiskers indicate $\pm$ 1.5 $\times$ interquartile range.

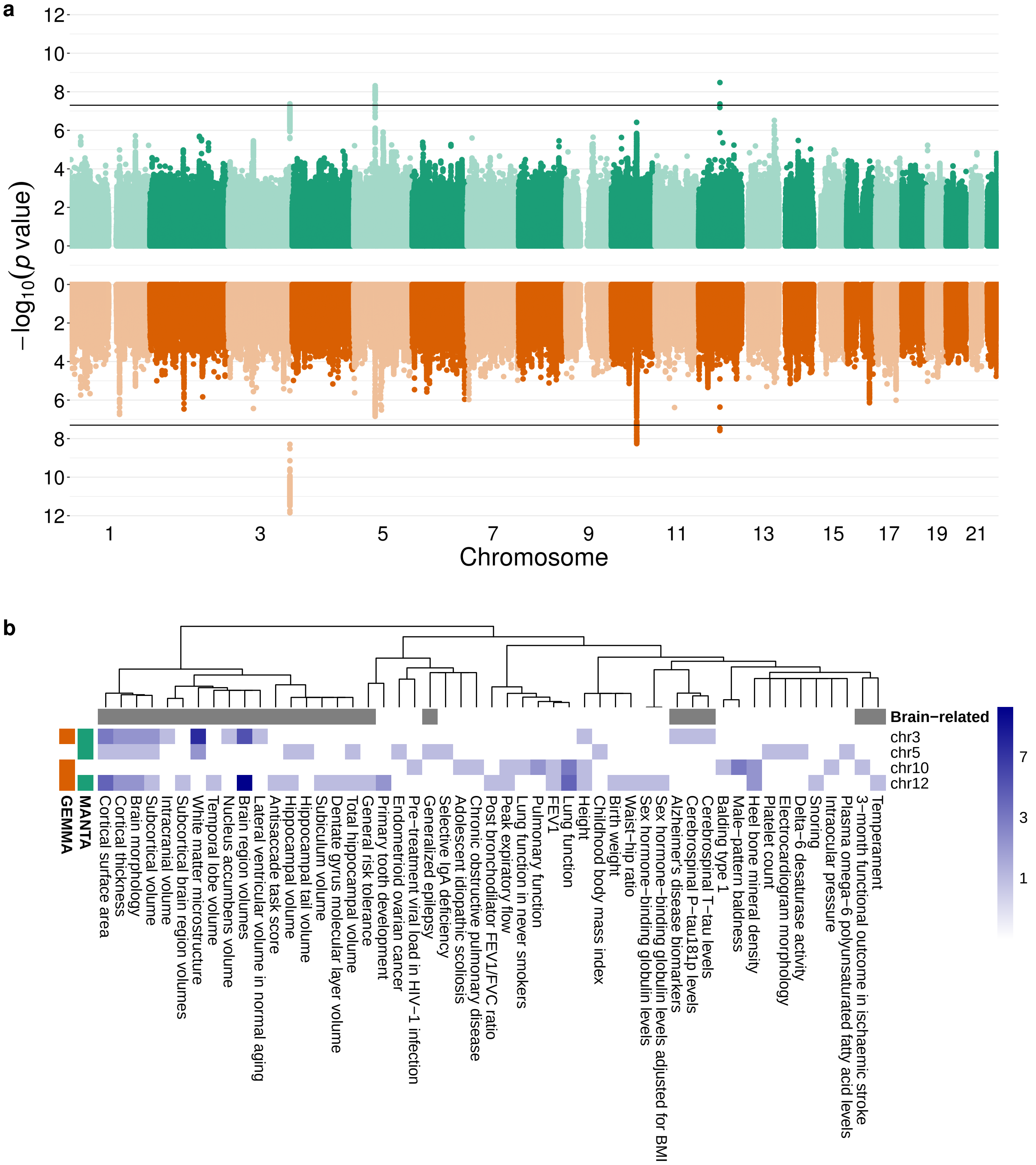

Fig. S32. a) Miami plot showing the $-\log_{10} p$ value of association between genetic variants and the volumes of 12 hippocampal subfields, obtained with MANTA (green) and GEMMA (orange). The horizontal black lines mark the genome-wide significance threshold selected ($5 \cdot 10^{-8}$). **b)** Heatmap representing the number of SNPs in the GWAS Catalog associated with a given trait (columns), that overlap each of the four loci identified (rows). Hierarchical clustering of GWAS traits is based on a distance matrix built upon their semantic similarity (see Methods). Brain-related traits are highlihgted in gray.

Supplementary Note 1: Mathematical proofs

Consider the matrices **Y** and **X**, defined in the main text. Following Anderson, $\mathbf{D} = (d_{ij})$ is the inter-distances matrix between individuals, $\mathbf{A} = (a_{ij}) = \left(-\frac{1}{2}d_{ij}^2\right)$, then **G** in (9) is the matrix introduced by Gower[5]:

$$\mathbf{G} = \left(\mathbf{I} - \frac{1}{n}\mathbf{1}\mathbf{1}^{\mathrm{T}}\right)\mathbf{A}\left(\mathbf{I} - \frac{1}{n}\mathbf{1}\mathbf{1}^{\mathrm{T}}\right)$$

In this general context, Anderson defines the pseudo-F statistic as:

$$\tilde{\mathrm{F}} = \frac{\mathrm{tr}(\mathbf{HGH})/\mathrm{rank}(\mathbf{H})}{\mathrm{tr}((\mathbf{I}-\mathbf{H})\mathbf{G}(\mathbf{I}-\mathbf{H}))/\mathrm{rank}(\mathbf{I}-\mathbf{H})} \tag{9}$$

Note that equation numbers continue from the main text.

Lemma 1. If **Y** is a centered-column matrix, and **D** is computed using the Euclidean distance, the Anderson statistic in (9) can be expressed as:

$$\tilde{\mathrm{F}} = \frac{\mathrm{tr}(\mathbf{Y}^{\mathrm{T}}\mathbf{HY})/\mathrm{rank}(\mathbf{H})}{\mathrm{tr}(\mathbf{Y}^{\mathrm{T}}(\mathbf{I}-\mathbf{H})\mathbf{Y})/\mathrm{rank}(\mathbf{I}-\mathbf{H})}$$

Proof. If $\mathbf{Y} = (y_{ij})$, any column mean $\bar{\mathbf{y}}_{\cdot j} = 0$. With the Euclidean distance and the definition of **G** it is straightforward to obtain:

$$\mathbf{G} = \mathbf{YY}^{\mathrm{T}}$$

The result is obtained combining the properties of the trace of the matrix product and the idempotence of the hat matrix **H**. □

Bai et al.[6] showed general results on the asymptotics of the m-estimation in the multivariate regression field. For our purposes, the main result in[6] is Theorem 2.4, which in our context can be simplified to:

Theorem 1. *Assume in model (1) the rows of* **E** *being i.i.d. with the same covariance matrix* Σ*. Its eigenvalue decomposition is* $\Sigma = \boldsymbol{P}\boldsymbol{\Lambda}\boldsymbol{P}^T$*,* $\boldsymbol{\Lambda} = diag(\lambda_j)$*,* $vec(\boldsymbol{\beta}) = \boldsymbol{\beta}_v$ *is the vectorized form of the* $\boldsymbol{\beta}$ *parameter matrix and* $\hat{\boldsymbol{\beta}}_v$ *is the vector of corresponding estimates. Under mild regularity conditions of the design matrix, the following result holds:*

$$(\hat{\boldsymbol{\beta}}_v - \boldsymbol{\beta}_v)^T(\boldsymbol{P}\boldsymbol{P}^T) \otimes (\boldsymbol{X}^T\boldsymbol{X})(\hat{\boldsymbol{\beta}}_v - \boldsymbol{\beta}_v) \xrightarrow{d} \sum_{j=1}^{q} \lambda_j \chi_j^2(p) \tag{10}$$

where $\chi_j^2(p)$ *is a collection of* q *independent chi-square variables with* p *degrees of freedom,* $\otimes$ *denoting the Kronecker product of two matrices.*

Proof. The complete proof can be found in[6], however, their notation has some differences with respect to the usual MMR notation adopted here. To help the reading of[6], we translate some symbols and provide details of the most important matrices. First, model in[6] is stated as:

$$\mathbf{Y}_i = \mathbf{X}_{i_B}^{\mathrm{T}}\boldsymbol{\beta} + \mathbf{E}_i \qquad i = 1, \cdots, n$$

$\mathbf{E}_i$ stands for a i.i.d. q-vector of errors, $\mathbf{X}_{i_B}$ is a $m \times q$ design matrix. Some other noticeable differences between notations are:

1. Bai's model equates the response of an *individual* i sample, while model (1) stands for the full sample of n individuals.

2. Bai's m stands for the dimension of $\boldsymbol{\beta}$, then, $m = p \times q$.

Denoting in model (1) row i of $\mathbf{X}$ as $\mathbf{X}_i$ (without B), both design matrices are related by:

$$\mathbf{X}_{i_B}^{\mathrm{T}} = \mathbf{I}_q \otimes \mathbf{X}_i$$

The mild regularity conditions described in the proposition refer specifically to condition (M6) in[6], that is, if $\mathbf{S}_n = \mathbf{X}_{1_B}\mathbf{X}_{1_B}^{\mathrm{T}} + \cdots + \mathbf{X}_{n_B}\mathbf{X}_{n_B}^{\mathrm{T}}$ then $\mathbf{S}_n$ must be non-singular for $n \geq n_0$ and

$$d_n^2 = \max_{1 \leq i \leq n} tr(\mathbf{X}_{i_B} \boldsymbol{S}_n^{-1} \mathbf{X}_{i_B}^{\mathrm{T}}) \to 0 \qquad as \quad n \to \infty$$

which can be interpreted as the individual design matrix having a leverage tending to zero, something that seems reasonable in practice. The remaining conditions (M1) to (M5) in[6] are trivially satisfied here. Finally, the auxiliary matrices $\mathbf{K}_n$ and $\mathbf{T}_n$ in the proof in[6] are, respectively:

$$\mathbf{K}_n = \mathbf{I}_q \otimes (\mathbf{X}^{\mathrm{T}}\mathbf{X})$$

$$\mathbf{T}_n = \boldsymbol{\Sigma} \otimes (\mathbf{X}^{\mathrm{T}}\mathbf{X})$$

$\square$

The covariance structure of the data in the first assumption of the theorem implies a global covariance matrix equal to $cov(vec(\mathbf{E})) = \mathbf{I} \otimes \boldsymbol{\Sigma}$ ($\mathbf{I}$ is the $n \times n$ identity matrix).

Under the omnibus null hypothesis, $\boldsymbol{\beta}_{\mathrm{v}} = \mathbf{0}$, the limiting distribution in (10) allows to obtain the trace in the numerator of the test statistic in (4):

$$\mathrm{tr}(\mathbf{Y}^{\mathrm{T}}\mathbf{H}\mathbf{Y}) = \hat{\boldsymbol{\beta}}_{\mathrm{v}}^{\mathrm{T}}(\mathbf{P} \otimes (\mathbf{X}^{\mathrm{T}}\mathbf{X})^{\frac{1}{2}})(\mathbf{P}^{\mathrm{T}} \otimes (\mathbf{X}^{\mathrm{T}}\mathbf{X})^{\frac{1}{2}})\hat{\boldsymbol{\beta}}_{\mathrm{v}} \xrightarrow{d} \sum_{j=1}^{q} \lambda_j \chi_j^2(p)$$

Consider now a null hypothesis where the parameters from $p_0 + 1$ to p are zero for all the q dimensions, that is, a hypothesis on a subset of the $p \times q$ possible parameters. Under this null hypothesis, for any single column of $\mathbf{Y}$, the corresponding column in matrix $\boldsymbol{\beta}$ in equation (1) will be multiplied by:

$$\mathbf{R} = \left(\mathbf{0}_{(p-p_0) \times p_0}\, , \mathbf{I}_{p-p_0}\right)$$

And joining all the dimensions we have the matrix $\mathbf{R}_{\mathrm{v}}$:

$$\mathbf{R}_{\mathrm{v}} = \mathbf{I}_q \otimes \mathbf{R}$$

which allows to express synthetically the hypothesis in the following Lemma, that specifies the limiting distribution of the Anderson's test for any subset of parameters.

Lemma 2. Assume a null hypothesis where all the parameters from columns p_0+1 to p in (1) are zero for all the q dimensions, that is:

$$\mathbf{R}_\mathrm{v}\,\boldsymbol{\beta}_\mathrm{v} = \mathbf{0}$$

then the numerator of the statistic in (2) converges in law to:

$$\mathrm{tr}\left\{\mathbf{Y}^\mathrm{T}\left(\mathbf{X}(\mathbf{X}^\mathrm{T}\mathbf{X})^{-1}\mathbf{X}^\mathrm{T} - \mathbf{X}_0(\mathbf{X}_0^\mathrm{T}\mathbf{X})_0^{-1}\mathbf{X}_0^\mathrm{T}\right)\mathbf{Y}\right\} \xrightarrow{d} \sum_{j=1}^{q}\lambda_j\chi_j^2(p-p_0)$$

Proof. The demonstration has three parts. First, consider the design matrix partitioned in two boxes $\mathbf{X} = (\mathbf{X}_0, \mathbf{X}_1)$ where $\mathbf{X}_0$ corresponds to $\mathbf{X}$ without the columns associated with the subset of parameters assumed to be zero. Then, prove that $\mathbf{H} - \mathbf{H}_0$ is an idempotent matrix.

Second, obtain the limiting distribution of $\mathbf{R}_\mathrm{v}\,(\hat{\boldsymbol{\beta}}_\mathrm{v} - \boldsymbol{\beta}_\mathrm{v})$ applying Theorem 1 and the *Product Limit Normal Rule*, and then derive the convergence in law of the following expression:

$$\left(\mathbf{I}_q \otimes \left(\mathbf{R}\,(\mathbf{X}^\mathrm{T}\mathbf{X})^{-1}\,\mathbf{R}^\mathrm{T}\right)^{-\frac{1}{2}}\right)\mathbf{R}_\mathrm{v}\,(\hat{\boldsymbol{\beta}}_\mathrm{v} - \boldsymbol{\beta}_\mathrm{v}) \xrightarrow{d} N(\mathbf{0},\,\boldsymbol{\Sigma}\otimes\mathbf{I}_{p-p_0})$$

Third, consider again the block partitioning of $\mathbf{X}$ in boxes $\mathbf{X} = (\mathbf{X}_0, \mathbf{X}_1)$ and prove that

$$\begin{aligned}\mathrm{tr}(\hat{\boldsymbol{\beta}}^\mathrm{T}\mathbf{X}^\mathrm{T}\mathbf{X}\hat{\boldsymbol{\beta}} - \hat{\boldsymbol{\beta}}_0^\mathrm{T}\mathbf{X}_0^\mathrm{T}\mathbf{X}_0\hat{\boldsymbol{\beta}}_0) &= \mathbf{y}_\mathrm{v}^\mathrm{T}\left(\mathbf{I}_q \otimes \left(\mathbf{X}(\mathbf{X}^\mathrm{T}\mathbf{X})^{-1}\mathbf{X}^\mathrm{T} - \mathbf{X}_0(\mathbf{X}_0^\mathrm{T}\mathbf{X}_0)^{-1}\mathbf{X}_0^\mathrm{T}\right)\right)\mathbf{y}_\mathrm{v} \\ &= \hat{\boldsymbol{\beta}}_\mathrm{v}^\mathrm{T}\,\mathbf{R}_\mathrm{v}^\mathrm{T}\left(\mathbf{I}_q \otimes (\mathbf{R}\,(\mathbf{X}^\mathrm{T}\mathbf{X})^{-1}\,\mathbf{R}^\mathrm{T})^{-\frac{1}{2}}\right)\left(\mathbf{I}_q \otimes (\mathbf{R}\,(\mathbf{X}^\mathrm{T}\mathbf{X})^{-1}\,\mathbf{R}^\mathrm{T})^{-\frac{1}{2}}\right)\mathbf{R}_\mathrm{v}\,\hat{\boldsymbol{\beta}}_\mathrm{v}\end{aligned}$$

Under the null hypothesis:

$$\left(\mathbf{P} \otimes \left(\mathbf{R}\,(\mathbf{X}^\mathrm{T}\mathbf{X})^{-1}\,\mathbf{R}^\mathrm{T}\right)^{-\frac{1}{2}}\right)\mathbf{R}_\mathrm{v}\,\hat{\beta}_\mathrm{v} \xrightarrow{d} N(\mathbf{0},\,\boldsymbol{\Lambda}\otimes\mathbf{I}_{p-p_0})$$

Because the limit covariance matrix is diagonal, all the $(p-p_0)\times q$ components are independent. The squared elements summed in the numerator correspond to squared univariate normals with zero mean and variance λ_j. Finally, group in $\chi_j^2(p-p_0)$ variables the components with identical eigenvalue. □

Denominators in (4) and (2) are identical to the sum of squared error terms of the simple regressions on each column of $\mathbf{Y}$. A well-known result of the OLS asymptotic properties states each of these terms converges in probability to the corresponding diagonal element of $\boldsymbol{\Sigma}$. The assumptions for such convergence are analogous to Bai's assumptions. Therefore, the denominator of the Anderson's statistic converges in probability to the trace of $\boldsymbol{\Sigma}$, that is, to $\sum_{j=1}^{q}\lambda_j$. Then, applying Lemma 2 and Slutsky's theorem, the limiting distribution of the statistic is obtained. In practice, probability tails can be computed considering only its numerator (the weights of the linear combination of chi-squares will be λ_j) or the pseudo-F ratio (weights will be $\lambda_j/\sum_{j=1}^{q}\lambda_j$).

As regards data transformations, it is straightforward to show that any transformation of $\mathbf{Y}$ that preserves the independence of the observations results in the same type of limiting distribution. Note, however, that the numerator of the test statistic, the eigenvalues of the residual covariance matrix and the resulting weighted sum of independent chi-square variables will differ with respect to the ones computed on the untransformed data.

Supplementary Note 2: Data generation in the simplex under H_0 and H_1

Given two points (i.e. vectors of proportions) in the $q-1$ simplex, $\boldsymbol{f}_1 = (f_{11}, \ldots, f_{1q})$ and $\boldsymbol{f}_2 = (f_{21}, \ldots, f_{2q})$, a problem of interest is to find the closest point in the simplex to $\boldsymbol{f}_1$, in the direction determined by $\boldsymbol{f}_2$, obtained by adding an amount Δ to f_{11}. We name this point $\boldsymbol{f}_1'$. In our context, we use the Hellinger distance to assess the dissimilarity between vectors of proportions, which is not Euclidean in its natural parametrization. Therefore, to find $\boldsymbol{f}_1'$ we need to respect the geometry induced by the distance.

The geometry of the Hellinger distance in the simplex is easier to visualize if we transform the proportions to their square root, $\boldsymbol{f}_i = (\sqrt{f_{i1}}, \ldots, \sqrt{f_{iq}})$, so that the vectors are located on the surface of the upper octant of a hypersphere of radius 1. In this surface, the shortest path between $\boldsymbol{f}_1$ and $\boldsymbol{f}_2$ is the geodesic given by:

$$f_{1j}' = \left\{ \sqrt{f_{1j}}\ \cos(d/2) + \frac{\sqrt{f_{2j}} - \sqrt{f_{1j}}\ \cos(\rho/2)}{\sin(\rho/2)}\ \sin(d/2) \right\}^2 \qquad j \in \{1, \ldots, q\} \tag{11}$$

where d is the distance traveled along the geodesic between $\boldsymbol{f}_1$ and $\boldsymbol{f_1'}$, and ρ the length of the arc of the hypersphere between $\boldsymbol{f_1}$ and $\boldsymbol{f_2}$, $\rho = 2\arccos(\sum_{j=1}^{q} \sqrt{f_{1j} f_{2j}})$.

After some straightforward algebra we can obtain the expression of the components $2 \ldots q$ along the geodesic satisfying $f_{11}' = f_{11} + \Delta$:

$$f_{1j}' = f_{1j} \left(1 - \frac{\Delta}{1 - f_{11}} \right) \quad j \in \{2, \ldots, q\} \tag{12}$$

During data generation in the multivariate proportion scenario, we employed equation (12) to generate $\boldsymbol{p'}$ from $\boldsymbol{p}$, that is, the multivariate mean under H_1 given the multivariate mean under H_0 and different values of Δ. Moreover, to actually generate random observations of vectors of proportions with certain variability, while ensuring that $E(\boldsymbol{f}_i) = \boldsymbol{p}$ (or, equivalently, $E(\boldsymbol{f}_i) = \boldsymbol{p'}$ under H_1), we proceeded as follows:

1. Generate a vector of q step sizes, $\boldsymbol{\delta}$, using any probability distribution, so that that $E(\delta_j) = 0$ and $Var(\delta_j) = \sigma_g^2$, for $j \in \{1, \ldots, q\}$. Specifically, we obtained $\delta_j \sim N(0, \sigma_g^2)$.
2. Select randomly the order in which the steps towards the simplex vertices $\{\boldsymbol{e}_1 = (1, 0, 0, \ldots, 0)$, $\boldsymbol{e}_2 = (0, 1, 0, \ldots, 0), \ldots$, $\boldsymbol{e}_q = (0, 0, \ldots, 1)\}$ are taken.
3. If $\boldsymbol{f}_i^{(0)} = \boldsymbol{p}$, for each $j = 1, \ldots q$, $\boldsymbol{f}_i^{(j)}$ is obtained from $\boldsymbol{f}_i^{(j-1)}$ advancing from $\boldsymbol{f}_i^{(j-1)}$ towards the vertex selected in the previous step, using equation (12) with $\Delta = \delta_j$. When $j = q$ the sample generated corresponds to the last step $\boldsymbol{f}_i = \boldsymbol{f}_i^{(q)}$.

Note that σ_g should be small enough so that $\sum_{j=1}^{q} f_{ij} = 1$, $\forall i \in \{1, \ldots, n\}$. In addition, the variances of the response variables generated, σ_{jj}^2, depend on the parameters σ_g, q, L and the probability distribution selected to generate δ_j. In practice, for a given probability distribution, we selected different values of σ_g to ensure $\overline{\boldsymbol{\sigma}_{jj}} = 0.03$ across different values of q and L.

References

[1] Duchesne, P. & Lafaye De Micheaux, P. Computing the distribution of quadratic forms: Further comparisons between the Liu–Tang–Zhang approximation and exact methods. *Computational Statistics & Data Analysis* **54**, 858–862 (2010).

[2] Mersmann, O. *microbenchmark: Accurate Timing Functions* (2019). `https://CRAN.R-project.org/package=microbenchmark`.

[3] McArtor, D. B., Lubke, G. H. & Bergeman, C. S. Extending multivariate distance matrix regression with an effect size measure and the asymptotic null distribution of the test statistic. *Psychometrika* **82**, 1052–1077 (2017).

[4] van der Meer, D. *et al.* Brain scans from 21,297 individuals reveal the genetic architecture of hippocampal subfield volumes. *Molecular Psychiatry* **25**, 3053–3065 (2018).

[5] Gower, J. C. Some distance properties of latent root and vector methods used in multivariate analysis. *Biometrika* **53**, 325–338 (1966).

[6] Bai, Z. D., Rao, C. R. & Wu, Y. M-Estimation of Multivariate Linear Regression Parameters Under a Convex Discrepancy Function. *Statistica Sinica* **2**, 237–254 (1992).
