# Supplementary material for: A fast non-parametric test of association for multiple traits
Source: Genome Biol. 2023 Oct 12;24:230. doi: 10.1186/s13059-023-03076-8 (PMC10571397; doi:10.1186/s13059-023-03076-8)
Supplement: Supplementary file 2 — Additional file 2. Review history. [file 13059_2023_3076_MOESM2_ESM.docx]

**Review history**

**First round of review**

**Reviewer 1**

Garrido-Martín et al. present a non-parametric test for multivariate responses that scales to hundreds of thousands of tests. With this method, new associations were found between genetic variants and alternative splicing or the volume of different brain sections. The methods are presented in great details. I found the simulations particularly helpful and comprehensive. The R package is also well documented, which further convinces me that this tool is going to be of broad utility in the field. I have one major comment around the first application, and a discussion suggestion to better understand the usability of the method.

1. My main concern is on the presentation of the "population-specific" QTL results. It's important to be careful with this topic as misinterpretation could have ramifications. There are multiple reasons why an association would be observed in one "population" and not another. For example, different LD structure, different power because the sample size is imbalanced or the allele frequencies are different, or, potentially more interesting, interaction with another genetic factor. At the minimum, I'd suggest that the author correct for some of these confounding effects and discuss the limitations. Optionally, it would be interesting to know if those factors could explain the examples highlighted in the text and figures, or explore interaction with other SNPs.
1. In GTEx Consortium et al. Science 2020 (cited in the manuscript), the population-biased eQTLs are found using tests that control "for differences in allele frequency, linkage disequilibrium (LD)". That article only describes associations after "applying stringent filters to remove differences potentially explained by LD or other artifacts". I couldn't find information about this kind of correction in this manuscript. It could remove many spurious associations.
2. In the KLK5 gene example, rs2739412 and SNPs nearby seems to be correlated differently in EUR vs AFR populations (e.g. using https://analysistools.cancer.gov/LDlink/?tab=ldproxy). More SNPs are in LD in EUR populations, which is the population where an association was detected. Were there other SNPs nearby that were associated in the AA population?
3. Could the unbalanced sample size (715 vs 103) explain the pb-sQTLs? What would happen if the EA were downsampled to 103 individuals? On a similar note, what is the proportion of pb-sQTLs that are EA-specific vs AA-specific? If it's balanced, it would be reassuring that the pb-sQTLs are not due to power/sample size imbalance.
4. The discussion does mention that the lack of association in a population could be used to avoid wrongly using this site when estimating genetic risk. It would be useful to know how many of the pb-sQTLs fall in that category, and how many are candidate for actual biological differences (i.e. after correcting/filtering for LD/power/AF confounders).
5. Finally, assuming the associated variant is causal (or tagging the causal variant in both populations), could it interact with the another SNP which might have different frequency between populations? To explore this, it could be interesting to test interaction between the associated SNP and nearby (or trans) SNPs. Acknowledging that it represents a substantial amount of work, this is just a suggestion to go further. It is much less important than the other points above.
2. A discussion of the curse of dimensionality would be helpful for future users. It's mentioned that "High error rates were found [..] when [..] the number of traits analyzed was relatively large." Some analyses were ran for up to 32 traits. Still, it's not clear how much power decreases when too many traits are added, or how many is too many.
3. Minor comments
1. The GitHub repositories are well documented. I appreciated the effort to share the different tools, including in pipeline form. I would only suggest tagging the version used for the analysis in the manuscript to further increase reproducibility. Also, having special features in a separate "interaction" branch could make it harder to find. Is there a reason why this mode is not part of the main branch/release?
2. In the second application, the absolute volume of subfields were used without normalization as far as I understood, but the total volume was added as a covariate in the model. Why not directly normalizing the volumes by the total, similar to what is done for the transcript expressions in the first application?
3. The legend of several boxplots mentions that "for visualization purposes, outliers are not shown". Is it because there are a lot of outliers? Or just a few but that stretch the axis a lot? If the former, it would be worth showing them. If the latter, maybe the authors could consider using winsorization (e.g. implemented in DescTools R package) to include them.

**Reviewer 2**

The authors present a multivariate test of genetic association, based on an asymptotic approximation to P values. The work is clearly explained, and such an approximation necessary as producing enough permutations to estimate the low p values is clearly infeasible. However, I would raise the following issues

My main issue is that all comparisons with other methods are based only on the analysis of simulations. While these can be beneficial, I think it is important to demonstrate that the method also performs well in the wild, does GEMMA find more associations when applied to the hippocampal data. In addition to this the issue of population stratification is only discussed in one paragraph. But the main justification for methods such as GEMMA is that they remove false positives due to population stratification, so I think this would be worth exploring in the real data, whether the P values produced show high inflation, if GEMMA is removing signals due to pop strat.

I did not understand why the splicing analysis look at interactions with ancestry, rather than splice QTLs themselves. While a package that can look multivariately at interactions would be beneficial, this paper is not being sold in this way. It means also that comparisons with other methods is not possible. For a paper selling a method it seems better to do the obviously analysis first, either joint analysis of multiple splicing phenotypes or cross tissue eQTL discovery, doing something more esoteric leaves the reader wondering why this was.

Finally, while computational time and resource usage is discussed, I think this could benefit from some context as it is not clear to me this is a big problem for GWAS studies. BOLT-LMM and GEMMA have been run on the whole of UK Biobank, so if there is a computational hurdle, it doesn't seem insurmountable. Is this not true for multivariate analysis of sufficient phenotypes?

In short, I found this a well written and interesting paper. I would like to see how this method compares on real world data.

**Authors’ response to reviewers**

# Reviewer #1

Garrido-Martín et al. present a non-parametric test for multivariate responses that scales to hundreds of thousands of tests. With this method, new associations were found between genetic variants and alternative splicing or the volume of different brain sections. The methods are presented in great details. I found the simulations particularly helpful and comprehensive. The R package is also well documented, which further convinces me that this tool is going to be of broad utility in the field. I have one major comment around the first application, and a discussion suggestion to better understand the usability of the method.

1.1. My main concern is on the presentation of the "population-specific” QTL results. It's important to be careful with this topic as misinterpretation could have ramifications. There are multiple reasons why an association would be observed in one "population" and not another. For example, different LD structure, different power because the sample size is imbalanced or the allele frequencies are different, or, potentially more interesting, interaction with another genetic factor. At the minimum, I'd suggest that the author correct for some of these confounding effects and discuss the limitations. Optionally, it would be interesting to know if those factors could explain the examples highlighted in the text and figures, or explore interaction with other SNPs.

We thank the reviewer for raising this important and sensitive issue. We agree with their concerns, and have performed additional checks and updated the manuscript accordingly. Please note that all the line numbers and figures referred below, unless stated otherwise, correspond to the new version of the manuscript.

1.1.1. In GTEx Consortium et al. Science 2020 (cited in the manuscript), the population-biased eQTLs are found using tests that control "for differences in allele frequency, linkage disequilibrium (LD)". That article only describes associations after "applying stringent filters to remove differences potentially explained by LD or other artifacts". I couldn't find information about this kind of correction in this manuscript. It could remove many spurious associations.

The GTEx Consortium (Science, 2020) implements a very different approach to identify pb-eQTLs. They do not use an interaction test. Instead, they first perform standard eQTL mapping in the whole cohort, and then, separately, in the african american (AA) and european american (EA) subcohorts. So they end up with 3 eQTL sets. As they do not perform a statistical test to specifically assess the interaction between the genotype and the population, they need to apply subsequent filters to discard all the variants that do not qualify as pb-eQTLs according to their definition. They do this through a decision tree-like strategy based on several custom rules, involving LD between top variants, p-value thresholds and allelic fold change information (GTEx Consortium paper, [Supplementar](https://www.ncbi.nlm.nih.gov/pmc/articles/PMC7737656/bin/NIHMS1651674-supplement-Supplementary_Materials.pdf)y [material](https://www.ncbi.nlm.nih.gov/pmc/articles/PMC7737656/bin/NIHMS1651674-supplement-Supplementary_Materials.pdf) pages 28-29, section “Population-biased cis-eQTL mapping”). They refer to this when they claim “applying stringent filters to remove differences potentially explained by LD or other artifacts''

Regarding MAF. Rather than *allele* frequencies, what matters to us are the related *genotype* frequencies. Small MAFs (particularly in the smaller AA cohort) can result in very few individuals with a given genotype, which could lead to spurious associations. The GTEx Consortium (Science, 2020) accounts for this by setting an “*additional filter of MAF > 10% in both groups*”. We believe that this is still too permissive. E.g. consider a SNP with MAF 0.1. For n = 86 (AA cohort size), the expected Hardy-Weinberg frequency of minor allele homozygous genotypes is 0.1^2^ = 0.01. That is, we expect only 86*0.01 ≈ 1 individual with this genotype in the AA cohort. In our work, we were more stringent, and “*required at least*

*10 samples per observed level of the interaction term*” (*Results* section, lines 285-286), ensuring that all the parameters can be estimated with at least 10 samples (this is further reassuring given the asymptotic nature of our approach). This implies, in practice, analyzing variants with larger MAFs (in our case the median MAF of tested variants was 0.3). Nevertheless, if differences in MAF between populations were biasing pb-sQTL mapping, we should observe different distributions of these values between pb-sQTLs and non-pb-sQTLs. However, no significant differences were found (Fig. R1). Even when focusing on the largest differences overall (|ΔMAF| > 0.3, either if MAF_AA_ < MAF_EA_ or MAF_AA_ > MAF_EA_) the percentage of pb-sQTLs (2.4%) and no pb-sQTLs (2.2%) was not significantly different (𝜒^2^ test p-value 0.28).

**Figure R1.** For all the variants tested in our analysis, we computed their MAF in the EA and AA cohorts. Then we calculated the difference, ΔMAF = MAF_EA_ - MAF_AA_. We represent the distribution of

|ΔMAF| values for variants identified as significant pb-sQTLs and not, separately for cases where MAF_AA_ < MAF_EA_ (left) and where MAF_AA_ > MAF_EA_ (right). The p-values shown correspond to Wilcoxon Rank-Sum tests.

Regarding LD. Given that each SNP is tested independently within a different model, LD structure has no statistical effect on nominal p-values. However, it may affect the multiple testing correction if QTL mapping is performed separately in different cohorts, as in GTEx Consortium (Science, 2020). For example, let’s consider 100 SNPs that have been tested for association with the expression (or splicing) of a gene, separately in the AA and EA cohorts. Let’s have a look at the most extreme situation: the average LD (r^2^) among the SNPs is 0 in AA and 1 in EA. The *effective* number of tests in the AA analysis is 100, while in the EA analysis is 1. Hence, multiple testing correction (e.g. by eigenMT) will be much more stringent in AA. However, this is not the case of our work. We do not perform QTL mapping separately for each cohort. We assess significance of the interaction term (genotype x population) using the whole dataset, and multiple testing correction via eigenMT is based on the LD structure of the whole dataset (same data for QTL mapping and LD calculations, as recommended by eigenMT authors, [doi.org/10.1016/j.ajhg.2015.11.021](http://doi.org/10.1016/j.ajhg.2015.11.021)). We have a single list of p-values for each gene, and independently of the multiple testing threshold, even if it is a function of LD, it won't result in different significant tests for AA and EA. Hence we believe that LD is not a statistical confounder in our case. Finally, if differences in LD between populations were biasing pb-sQTL mapping, we should observe different distributions of these values between pb-sGenes and no pb-sGenes. However, no significant differences were found (Fig. R2). Even when focusing on the largest differences overall (|Δ mean(r^2^)| > 0.3, either if LD_AA_ < LD_EA_ or LD_AA_ > LD_EA_), the percentage of pb-sGenes (3.4%) and no pb-sGenes (3.7%) was not significantly different (𝜒^2^ test p-value 0.66).

**Figure R2.** For each tested gene, we computed the average r^2^ between all pairs of variants tested for association with the gene, separately in the EA and AA cohorts. Then we calculated the difference, Δmean(r^2^) = mean(r^2^)_EA_ - mean(r^2^)_AA_. We represent the distribution of |Δmean(r^2^)| values for variants identified and not as significant pb-sQTLs, separately for cases where mean(r^2^)_AA_ < mean(r^2^)_EA_ (left) and where mean(r^2^)_AA_ > mean(r^2^)_EA_ (right). The p-values shown correspond to Wilcoxon Rank-Sum tests.

🧾 In the revised version of the manuscript, we briefly discuss MAF and LD as potential biases in pb-sQTL discovery, and include the analyses above and Figs. R1 and R2 as new Supp. Fig. S19a,b (see *Results* section, lines 275-280; *Methods* section, lines 292-301, *Discussion* section, lines 447-450).

1.1.2. In the KLK5 gene example, rs2739412 and SNPs nearby seems to be correlated differently	in	EUR	vs	AFR	populations	(e.g.	using https://analysistools.cancer.gov/LDlink/?tab=ldproxy). More SNPs are in LD in EUR

populations, which is the population where an association was detected. Were there other SNPs nearby that were associated in the AA population?

As pointed out above, LD does not seem a statistical confounder in our analysis. In the particular case of this example, note also that EUR and AFR reference populations pointed out may not match exactly our EA and AA cohorts, and that not all the variants in the region were tested for association. We computed LD (r^2^) between rs2739412 and the other 22 variants tested for association with KLK5 splicing, separately in our EA and AA cohorts. We observed that, in the vast majority of cases, a given variant pair tended to lay in the same LD range in both populations (often displaying either very high or very low LD, Fig. R3).

**Figure R3.** Variant pairs (formed by rs2739412 and a second variant tested for association with KLK5 splicing) are assigned to cells based on their LD (r^2^) range in EA (rows) and AA (columns) cohorts.

Regarding the second question on “association in the AA population”, please note that our approach does not provide information about the size of the effect, neither globally nor for each population. A significant p-value can only be interpreted as “the variant affects the splicing of the gene *differently* in EA and AA” (see point 1.1.3 below for further discussion). Still, if we look at MD values (taking into account the caveats also described below) these are substantially larger in the EA population (on average 0.16) than in the AA population (on average 0.04), suggesting that the sQTL effect is only present in the EA cohort.

Note also that the KLK5 example is further supported by the splice junction analysis.

1.1.3. Could the unbalanced sample size (715 vs 103) explain the pb-sQTLs? What would happen if the EA were downsampled to 103 individuals? On a similar note, what is the proportion of pb-sQTLs that are EA-specific vs AA-specific? If it's balanced, it would be reassuring that the pb-sQTLs are not due to power/sample size imbalance.

We acknowledge that sample size imbalance is a limitation of our pb-sQTL analysis. In our context, unless assumptions such as homoskedasticity are violated (see Fig. 2c), and given that we require at least 10 samples per level of the interaction to perform the testing, sample size imbalance will not increase type I error. However, It may substantially affect power. As the reviewer points out, the question is whether it affects power to detect effects differently in the two populations.

To investigate this further we have simulated the following scenario: a gene with three splicing isoforms, measured in n = 1000 individuals; a factor with two levels (i.e. the two populations), with decreasing proportions of the less represented one (i.e. AA, from 0.5 to 0.1); and a second factor with three levels (i.e. a binomial SNP with MAF 0.3, set as the median MAF in our real dataset). We simulated splicing ratios as in the *vectors of proportions* scenario (*Methods* section, lines 83-96). We simulated a completely population-*specific* sQTL, that is, an additive effect (of arbitrary size, 𝛿 = 0.005) in one of the populations and no effect in the other. A large total sample size was selected to ensure a minimum of 10 individuals per level of the interaction term (the expected number of individuals in the AA/minor allele level of the interaction is 1000·0.1·0.3^2^ = 9, we sampled until the requisite was fulfilled). We tested the interaction term with MANTA in 1000 simulations, assessing models analogous to the one in the real pb-sQTL analysis. Finally we computed power (number of tests significant at ɑ < 0.05 after Bonferroni correction). To estimate the variability of power measurements, we repeated all the previous procedures (data generation, simulations, power assessment) 100 times. Results are shown in Fig. R4a. We observed that power consistently decreases with the level of sample size imbalance, as expected. However, this decrease is identical for AA- and EA-specific effects. Hence, sample imbalance does not seem to bias pb-sQTL discovery towards EA-specific or AA-specific sQTLs. We also simulated a scenario without sQTL effect (𝛿 = 0) in any population to show the expected absence of inflated type I error when there is sample size imbalance (Fig. R4b).

# a) b)

**Figure R4.** a) Power to detect a population-specific effect of arbitrary size on alternative splicing of a gene with 3 isoforms. The x-axis shows the percentage of individuals belonging to the (simulated) AA cohort. Colors represent the results corresponding to the scenario where the sQTL effect is present in the AA (red) or in the EA (blue) population -and absent in the other population-. Note the absolute power is not relevant given that the effect size is arbitrary. b) Type I error observed in a simulation analogous to the one displayed in panel a) in the absence of sQTL effect in any population (𝛿 = 0). x-axis shows the percentage of individuals belonging to the (simulated) AA cohort.

🧾 In the revised version of the manuscript, we discuss the limitations of sample size imbalance and include the results of the power simulation, with Fig. R4a as new Supp. Fig. S20. (*Results* section, lines 288-290; *Discussion* section, lines 451-452; *Methods* section, lines 302-312)

Regarding EA- or AA-*specific* sQTLs. Please note that in our interaction analysis, a significant p-value can only be interpreted as “the variant affects the splicing of the gene *differently* in EA and AA”. Our method does not provide information about the size of the effect (as we acknowledged in the *Discussion* section lines 501-503), neither globally nor for each population. Multivariate phenotypes can be challenging in this regard: e.g. in a gene with 3 transcripts, a SNP can affect the expression of transcripts *t_1_* and *t_2_* in EA (but not in AA), and transcripts *t_2_* and *t_3_* in AA (but not in EA), with a different effect size. This becomes more complex with many splicing isoforms. Even if the effect is larger in one of the populations, we cannot consider a multi-trait effect like the previous *population-specific*, but rather *population-biased*. We could only distinguish a scenario where there is a *different* effect in each population (as we just described) *vs.* a scenario where the *same* effect is present in one population and absent in the other (i.e. *specificity*) by looking at the transcript abundances on a case-by-case basis.

In previous works with splicing QTLs, we used the MD value (absolute maximum difference in adjusted mean transcript relative expression -corresponding to the two transcripts that change the most relative to each other- between genotype groups) as a univariate proxy for the size of the effect ([Garrido-Martín et al., Nat. Comms. 2021](https://doi.org/10.1038/s41467-020-20578-2)). MD takes values in the interval [0, 1], but in practice usual MD values belong to [0.01, 0.4]. As a general rule, we consider MD values < 0.1 as small effect sizes, MD values between 0.1 and 0.2 as moderate effect sizes, and MD values greater than 0.2 as large effect sizes. With sQTLseekeR2.int we computed MD values for pb-sQTLs, separately for AA and EA. However, a direct comparison of these values is not straightforward, given that the two transcripts involved in each calculation in most cases do not coincide.

Still, in order to answer the reviewer's question, we defined a set of *pseudo* population-specific sQTLs by setting different thresholds on MD values. E.g., we defined pseudo EA-specific sQTLs as those pb-sQTLs having a medium/large MD value in EA (MD_EA_

> 0.1), a small MD value in AA (MD_AA_ < 0.1), and a large difference between both, MD_EA_ - MD_AA_ > 0.1. Even if it could happen that MD_AA_ and MD_EA_ are computed using different transcripts, meaning they could reflect different effects, we require MD_AA_ to be small enough so that in practice this can be dismissed. We defined pseudo AA-specific sQTLs analogously. As MD is tissue-dependent, we considered all the tissue/pb-sGene/pb-sQTL trios identified as significant (10,255). Of those, 1,466 were pseudo AA-specific and 1,326 were pseudo EA-specific, according to our definitions. As can be seen, both numbers are very similar, and do not reflect the sample size imbalance in favor of the EA cohort.

🧾 In the revised version of the manuscript, we further clarify the interpretation of the interaction and the fact that our method does not provide effect size estimates (*Methods* section, lines 268-268). We also provide MD values computed in AA and EA populations, as well as the number of coincident transcripts used in their calculation (0, 1 or 2), with the summary statistics of the pb-sQTL analysis.

Finally, for each tissue, we randomly downsampled the EA cohort so that the number of EA and AA individuals was the same, and ran again pb-sQTL mapping for tissues with total sample size ≥ 100. We repeated this 10 times, generating 10 pb-sQTLs sets. We computed the average fraction of tested genes identified as pb-sGenes in each downsampled dataset (

𝑓_i_) and in the original dataset (𝑓_0_). The ratio 𝑓_0_/𝑓_i_ was not correlated with the AA fraction in the original dataset (Fig. R5, Pearson *r =* -0.09, p-value 0.78), suggesting that total sample size and tissue biology, rather than sample imbalance, are the major drivers of differences in pb-sQTL discovery among tissues (see Fig. R5 below).

**Figure R5.** For each downsampled tissue (with sample size ≥ 100), we represent the fraction of AA individuals (x-axis) *vs* the ratio between the fraction of tested genes identified as pb-sGenes in each downsampled dataset (on average, 𝑓_i_) and in the original dataset (𝑓_0_) (y-axis).

To evaluate replication of the full-size results in the downsampled datasets, we should also take into account the impact in power of the substantial drop in total sample size (on average across tissues, downsampling supposed a 75% decrease with respect to the original sample sizes). We computed how many tissue/pb-sGene pairs identified in the original dataset under a given p-value threshold were also identified in any of the downsampling analyses. As can be seen in Fig. R6, over 80% of the most significant pairs were also identified in the downsampling analyses, suggesting again that our pb-sQTLs are not due to sample size imbalance.

**Figure R6.** Percentage of replicated pb-sGene/tissue pairs in the downsampled analysis for different p-value thresholds set in the complete dataset analysis.

Moreover, if we focus on trios tissue/pb-sGene/pb-sQTL defined as pseudo EA- or pseudo AA- specific based on MD values (as described above), identified in the original dataset under a reasonable p-value threshold to account for the power loss due to downsampling (e.g. p-value < 1e-6), they are replicated in the downsampling analyses in similar proportions: 59% vs 53%, respectively (𝜒^2^ test p-value 0.45).

1.1.4. The discussion does mention that the lack of association in a population could be used to avoid wrongly using this site when estimating genetic risk. It would be useful to know how many of the pb-sQTLs fall in that category, and how many are candidate for actual biological differences (i.e. after correcting/filtering for LD/power/AF confounders).

In the discussion we wrote “*This is an example of how the European bias of genetic association studies could lead to inaccurate risk assessment in non-European populations, and strongly argues for increasing the diversity of such studies, by including individuals of under-represented ancestries''*. We did not mean that our pb-sQTLs could be used to improve the estimation of genetic risk, but rather that they provide an additional molecular justification to compute polygenic risk scores (PRSs) either stratified by ancestry or incorporating ancestry information. It is widely acknowledged that PRSs display poor generalizability across diverse ancestries, and often present reduced accuracy in non-European populations ([Sirugo G. et al.](https://doi.org/10.1016/j.cell.2019.02.048) *[Cell](https://doi.org/10.1016/j.cell.2019.02.048)* [2019](https://doi.org/10.1016/j.cell.2019.02.048); [Duncan L. et al. *Nat. Commun.* 2019](https://doi.org/10.1038/s41467-019-11112-0); [Martin A.R. et al. *Nat. Genet.* 2019](https://doi.org/10.1038/s41588-019-0379-x); [Wang Y. et al. *Annu. Rev. Biomed. Data Sci.* 2022](https://doi.org/10.1146/annurev-biodatasci-111721-074830)). Indeed, several methods to build PRSs incorporating ancestry information have been proposed ([Coram, M.A. et al. *AJHG* 2017](https://doi.org/10.1016/j.ajhg.2017.06.015), [Cai, M. et al, *AJHG* 2021](https://doi.org/10.1016/j.ajhg.2017.06.015); [Weissbrod, O. et al.](https://doi.org/10.1038/s41588-022-01036-9) [*Nat. Genet.* 2022](https://doi.org/10.1038/s41588-022-01036-9)). It is unclear how pb-QTLs could be used to improve the prediction of risk scores, if they could even be. Most likely they would add very little to the estimation of PRSs using methods that already incorporate ancestry information.

In addition, in our case, as clarified above, we do not assess the significance of the association in each population, but rather the significance of the interaction term. The presence of a significant interaction does not provide information of the size of the effect, nor whether it is or not population-specific. Hence, a significant interaction should be considered just a first step that needs to be followed-up to determine which are the actual differences between populations (which isoforms change, to which extent, etc). Just to provide some estimates, our analysis of MD values (see point 1.1.3. above) suggests that approximately (1,466+1,326)/10,255*100 = 27.2% of the tissue/gene/variant trios tested are pseudo population-specific. This is a relatively large number that would justify the calculation of ancestry-aware PRSs.

1.1.5. Finally, assuming the associated variant is causal (or tagging the causal variant in both populations), could it interact with another SNP which might have different frequency between populations? To explore this, it could be interesting to test interaction between the associated SNP and nearby (or trans) SNPs. Acknowledging that it represents a substantial amount of work, this is just a suggestion to go further. It is much less important than the other points above.

We agree with the reviewer that exploring SNP-SNP (i.e. epistasic) interactions could be an interesting follow-up. There are, however, several limitations that would hinder this analysis, such as LD (i.e. two variants in high LD would become two highly associated predictor variables in the MANTA model. This is equivalent to “multicollinearity” in the case of quantitative predictors) and problems with empty (or with few samples) levels of the interaction (i.e. difficulty to find enough individuals that are e.g. homozygous for the minor allele in both variants ─even for relatively large MAFs─, and particularly if they are in low LD, which may be required, as has been pointed out). We should also take into account the increased computational burden of testing pairs. As the reviewer acknowledges, to perform a proper analysis (designing an efficient filtering strategy to account for all the previous while removing the smallest possible number of variants, testing interactions for all the resulting pairs, integration with the population-biased sQTL analysis, interpretation of the results) represents a large amount of work. In our view, many factors need to be taken into account and this should be considered a project by itself. Please note that the main goal of the examples described in our manuscript is to illustrate the applicability of our method in realistic scenarios, rather than to perform a comprehensive downstream analysis in each case. Still, assessing the effect of genetic interactions on multiple traits (either at molecular or at organismal level) is an interesting application of the fast interaction test that we have implemented in MANTA.

🧾 In the revised version of the manuscript, we mention the potential application of MANTA to investigate (pleiotropic) epistasis (*Discussion* section, lines 479-480).

1.2. A discussion of the curse of dimensionality would be helpful for future users. It's mentioned that "High error rates were found [..] when [..] the number of traits analyzed was relatively large." Some analyses were ran for up to 32 traits. Still, it's not clear how much power decreases when too many traits are added, or how many is too many.

The sentence mentioned by the reviewer is in the *Implementation* subsection of the *Methods* section, and it is a practical note that refers only to the GEMMA software (see also Supp. Figs. 10 and 16 and *Results* section, lines 212-217). With ‘error rates’, we meant ‘failure rates’, related to the previous sentence: ‘Numerical optimization in GEMMA failed [...] for a small fraction of the tests performed.’.

🧾 In the new version of our manuscript (*Methods* section, lines 234-235), we have rephrased to avoid confusion:

*Numerical optimization in GEMMA failed* [...] *for a small fraction of the tests performed. However, the number of failures was large in certain scenarios, e.g. when the trait distribution deviated substantially from multivariate normality or the number of traits analyzed was relatively large.*

Regarding the more general question on the impact of the number of traits, we already discussed this in the context of the asymptotic nature of our method (*Results* section, lines 146-156). We show that the key factor is the ratio #observations (n) / # traits (q). The accuracy of our p-values depends on this ratio. The number of traits for which we can accurately compute p-values depends essentially on the number of observations that we

have (let’s assume independent traits for simplicity, other situations with increasing degrees of correlation -in absolute value- between traits would be equivalent to scenarios with fewer independent traits). *Many* traits won’t be *too many* as long as we have *enough* sample size. We indeed give the users the practical rule of having ~20 observations per trait. With fewer observations per trait, our method will be most likely underpowered, but will not produce false positives (see Fig. 1b, Supp. Fig. 4). When we have enough sample size different scenarios may arise. For example, adding independent traits associated with the factor of interest increases power (see Supp. Fig. 10a). A related, but different question is how interpretable is an effect on many traits, but this is beyond the usage of any method.

Although not relevant for the discussion above, thanks to this comment, we have also identified a typo in the text: our running time (and RAM usage) simulations were run for a number of traits between 2 and **20** (not 32). This was an oversight and it is now corrected. In the Figure captions the number was already correct.

1.3. Minor comments
     1.3.1. The GitHub repositories are well documented. I appreciated the effort to share the different tools, including in pipeline form. I would only suggest tagging the version used for the analysis in the manuscript to further increase reproducibility. Also, having special features in a separate "interaction" branch could make it harder to find. Is there a reason why this mode is not part of the main branch/release?

We thank the reviewer for the suggestion. We now tag all the versions used in the manuscript. Regarding the second point, whereas MANTA can assess significance of any model, and it is production-ready either through the R package or the mvgwas-nf nextflow pipeline, sQTLseekeR2’s interaction mode was only implemented for a very specific model (Y ~ A + B + A:B), with some limitations (factor A categorical, with two levels, no other covariates in the same model, etc.). We aim at incorporating a full version of MANTA within the core computation of sQTLseekeR2. This is still work in progress but as soon as this feature is production-readly will be merged into the master branch.

1.3.2. In the second application, the absolute volume of subfields were used without normalization as far as I understood, but the total volume was added as a covariate in the model. Why not directly normalizing the volumes by the total, similar to what is done for the transcript expressions in the first application?

There were two reasons for this: i) to be consistent with previous studies (e.g. [van der Meer,](https://doi.org/10.1038/s41380-018-0262-7) [et al. Mol. Psych, 2018](https://doi.org/10.1038/s41380-018-0262-7)), as in the brain field it is relatively common to control for intracranial volume/whole brain volume or whole hippocampal volume by including it as a covariate; and

ii) we also wanted to illustrate the usefulness of our method in a scenario where traits are not proportions.

**1.3.2.** The legend of several boxplots mentions that "for visualization purposes, outliers are not shown". Is it because there are a lot of outliers? Or just a few but that stretch the axis a

lot? If the former, it would be worth showing them. If the latter, maybe the authors could consider using winsorization (e.g. implemented in DescTools R package) to include them.

Outliers were initially removed from old Figs. 4c, S24 and S28b (now Figs. 4c, S26 and S30b) because a few outliers stretched the y-axis and to better accommodate the information about the significance of pairwise tests. We agree with the reviewer that it is better to display them.

🧾 In the new version of the manuscript, when possible, we kept all of them (Fig. 4c and Supp. Fig. S30). Otherwise, we applied winsorization (Supp. Fig. 26), as suggested.

# Reviewer #2

The authors present a multivariate test of genetic association, based on an asymptotic approximation to P values. The work is clearly explained, and such an approximation necessary as producing enough permutations to estimate the low p values is clearly infeasible. However, I would raise the following issues

We thank the reviewer for the positive comments on our work. Please note that all the line numbers and figures referred below, unless stated otherwise, correspond to the new version of the manuscript.

2.1. My main issue is that all comparisons with other methods are based only on the analysis of simulations. While these can be beneficial, I think it is important to demonstrate that the method also performs well in the wild, does GEMMA find more associations when applied to the hippocampal data. In addition to this the issue of population stratification is only discussed in one paragraph. But the main justification for methods such as GEMMA is that they remove false positives due to population stratification, so I think this would be worth exploring in the real data, whether the P values produced show high inflation, if GEMMA is removing signals due to pop strat.

Before moving forward, we would like to mention that thanks to this comment we realized that we had not filtered out related individuals from the hippocampal subfield cohort prior to GWAS. Thus, we have repeated all the analyses after removing 560 individuals that displayed 1st, 2nd or 3rd degree relationships —i.e. KING ([Manichaikul A. et al.](https://doi.org/10.1093/bioinformatics/btq559) [*Bioinformatics* 2010](https://doi.org/10.1093/bioinformatics/btq559)) kinship coefficient > 0.8884— with others in the cohort. Total sample size after removing related individuals is 41,974 - 560 = 41,414. Overall, GWAS results after this filter are practically identical to the ones obtained before (-log_10_(p-value) Pearson correlation *r =* 0.991, See Fig. R7 below). This suggests that MANTA is relatively robust to the presence of a small fraction of related individuals (slightly over 1% in this case). 🧾 Still, in the new version of the manuscript, we report the new results and figures, corresponding to the dataset where related individuals have been removed.

**Figure R7.** Correlation between -log_10_(p-values) obtained by MANTA in the hippocampal subfield dataset before (x-axis) and after (y-axis) removing related individuals.

Regarding the reviewer's point on comparing MANTA vs GEMMA with real data. Running the multi-trait version of GEMMA on the complete hippocampal dataset (n = 41,414, q = 12, 8 million variants) is computationally infeasible (see point 2.3. below for further discussion regarding this). However, we agree with the reviewer that it is worth comparing MANTA and GEMMA in the context of real data. Hence, we randomly downsampled the hippocampal dataset to 10k individuals, and performed a GWAS using the two methods. Given the slightly different filtering strategies, we focused on the common set of tested variants. A Miami plot representing the results obtained is shown in Fig. R8.

**Figure R8.** Miami plot showing the results of the MANTA (green) and GEMMA (orange) GWAS.

Both MANTA and GEMMA identify 3 genome-wide significant loci (p-value < 5·10^-8^), two of them in common (Table R1). This is compared to the 41 significant loci that we identify when running MANTA on the entire cohort. The three loci identified by MANTA, but not the one identified only by GEMMA, were also found in the analysis of the complete cohort. Overlap with the GWAS Catalog shows that all four loci, except for the one identified only by GEMMA, contain variants previously related to hippocampal subfield volumes and other brain-related measurements (Fig R9). In particular, the locus in chromosome 5 identified only by MANTA replicates one of the loci associated with whole hippocampal volume in [Van der](https://www.nature.com/articles/s41380-018-0262-7) [Meer et al., 2018](https://www.nature.com/articles/s41380-018-0262-7), the largest univariate hippocampal subfield GWAS to date (n = 21,297), here with half of the sample size (n = 10,000).

**Table R1**. Genome-wide significant loci (p-value < 5·10^-8^) identified by MANTA and GEMMA in the downsampled hippocampal dataset (n = 10,000).

**Figure R9.** Overlap with the GWAS Catalog. The heatmap represents the number of SNPs in the Catalog associated with a given trait that overlap each of the four loci. Hierarchical clustering of GWAS traits is based on a distance matrix built upon their semantic similarity. Brain-related measurements are highlighted.

Overall, despite some differences, the results obtained with MANTA and GEMMA in the real hippocampal dataset are comparable. This is consistent with our simulation results, which show that both methods present similar power in the majority of scenarios, although in some cases the specific combination of genetic effects and trait-to-trait correlations may lead to differences in power between the two (*Results* section, lines 226-233). Please note, however, that i) the computational burden of obtaining these results is not comparable: from a few hours in the case of MANTA to 6 days in the case of GEMMA; and ii) the hippocampal subfield scenario is slightly favorable for GEMMA, as it does not deviate substantially from the multivariate normal assumption of the method (see point 2.3 below for further discussion regarding these topics).

🧾 In the new version of the manuscript, we have included all the analyses of the downsampled hippocampal dataset (*Results* section, lines 399-415; Methods section, lines 374-390, with Table R1 and Figs. R8 and R9 above as new Supp. Table S5 and Supp. Fig. 32a,b). As a side note, to perform this analysis we implemented gemma-nf, a highly parallelizable and reproducible pipeline to run GEMMA in computing clusters using Nextflow+Docker. We also make it available in the new version of the manuscript.

Regarding the reviewer’s issue on population stratification. Let’s distinguish between i) population stratification (large-scale, systematic differences in genetic ancestry among samples) and ii) other forms of relatedness, such as family structure (familial relatedness among samples) or cryptic relatedness (distant relatedness among samples with no known family relationships). Including the top principal components as covariates in GWAS successfully corrects for (i) if it is the only kind of structure present, although this approach is not appropriate in the presence of (ii) ([Pierce et al, *Nat. Review. Genetics*, 2010](https://doi.org/10.1038/nrg2813)). Indeed, (ii)

can only be accounted for via LMMs. In our simulations we actually show in detail (*Results* section lines 198-207, Supp. Fig. S8a) that MANTA+PC displays inflated type I errors in scenario (ii). Therefore, MANTA should not be used in this situation, and it is not an alternative to GEMMA (or other multivariate LMMs) in this case. All this is not only discussed in one paragraph, but rather mentioned in several points throughout the manuscript (*Methods* section lines 127-129, *Results* section lines 198-207*, Discussion* section, lines 487-496).

However, MANTA can be used instead of GEMMA when population structure is the only form of relatedness present. This is a common scenario (e.g. GTEx), or it can be achieved in a straightforward manner by discarding related individuals after genetic relationship inference via methods such as KING ([Manichaikul A. et al.](https://doi.org/10.1093/bioinformatics/btq559) *[Bioinformatics](https://doi.org/10.1093/bioinformatics/btq559)* [2010](https://doi.org/10.1093/bioinformatics/btq559)). In these cases, MANTA achieves comparable power and a dramatic improvement in running time (e.g. in the case of the analysis of the downsampled hippocampal dataset, from almost 6 days to a few hours). In addition, as MANTA is non-parametric, it should be preferred over GEMMA when the trait distribution deviates substantially from multivariate normality. It may occur that, in this situation, GEMMA’s numerical optimization fails (the software produces an error and does not generate any output) Indeed this is common e.g. with multivariate proportion data, see *Results* section lines 212-216 and *Methods* section lines 233-236)

In the analysis of the downsampled hippocampal dataset (where population stratification is present, but not other forms of relatedness), we did not observe a remarkable inflation of neither GEMMA nor MANTA p-values. As MANTA p-values do not come from a normal distribution, we used an equivalent to 𝜆_G_ computed on p-values: 𝜆_X_ = median(−log_10_(p_obs_))/median(−log_10_(p_exp_)) ). Genomic inflation factors were 𝜆_X_ = 1.02 for GEMMA and 𝜆_X_ = 1.07 for MANTA, respectively. GEMMA seems to do only slightly better correcting for population stratification than our 5 PCs (likely increasing the number of PCs would make both values even more similar).

🧾 In the new version of the manuscript, we incorporate to the *Discussion* the idea that relatedness can be inferred via methods such as KING, and that it is straightforward to discard related individuals prior to GWAS (*Discussion* section, lines 495-496). In addition, we report the inflation factors for MANTA and GEMMA in the downsampling analysis, showing that both are very similar and reflect no inflation (*Methods section*, lines 381-385)

2.2. I did not understand why the splicing analysis look at interactions with ancestry, rather than splice QTLs themselves. While a package that can look multivariately at interactions would be beneficial, this paper is not being sold in this way. It means also that comparisons with other methods is not possible. For a paper selling a method it seems better to do the obviously analysis first, either joint analysis of multiple splicing phenotypes or cross tissue eQTL discovery, doing something more esoteric leaves the reader wondering why this was.

[Anderson & Robinson (2003)](https://doi.org/10.1111/1467-842X.00285) proposed an asymptotic version of the PERMANOVA test, valid only when there is a single factor in the model (the SNP in our case). Any other scenario required permutations. We implemented this result in the sQTLseekeR R package and already used it to map ‘standard’ splicing QTLs (i.e. *splicing ratios ~ SNP_i_*) across human populations ([Monlong et al.,](https://doi.org/10.1038/ncomms5698) *[Nat. Commun.,](https://doi.org/10.1038/ncomms5698)* [2014](https://doi.org/10.1038/ncomms5698)) and tissues ([Garrido-Martín et al,](https://doi.org/10.1038/s41467-020-20578-2)

[*Nat. Comm*un., 2021](https://doi.org/10.1038/s41467-020-20578-2)), as the reviewer suggests. We mention this in the Background section (lines 59-63):

*Only for one-way fixed designs, Anderson and Robinson showed the asymptotic distribution of the numerator of the test statistic^27^. We used this result, implemented in sQTLseekeR, to identify genetic effects on alternative splicing across human populations^28^ and tissues^29^. Nevertheless, p value calculation in more complex designs still relied on permutations.*

In [Garrido-Martín et al, *Nat. Commun.*, 2021](https://doi.org/10.1038/s41467-020-20578-2), we also compared this approach with a second method: the univariate strategy based on linear regression of LeafCutter intron excision ratios employed by the [GTEx Consortium, *Science*, 2020](https://doi.org/10.1126/science.aaz1776).

In the current manuscript, however, we have derived a general asymptotic approximation for the PERMANOVA test, valid for any complex model including fixed factors, numerical covariates, interaction terms, nested effects, etc., that can be specified by the user. This is indeed one of the major contributions of our work (see *Background*, lines 64-65):

*To overcome this limitation, here we obtain the asymptotic distribution of the PERMANOVA test statistic for complex designs in the Euclidean distance case.*

Our approach provides the same result that the Anderson-Robinson approximation for a single factor, plus it can be employed in way more scenarios, so we decided to illustrate two of the situations that could not be analyzed without relying on permutations until now: i) identification of interaction effects (as in our first example: *splicing ratios ~ SNP_i_ +* population

*+ SNP_i_ * population*) and ii) inclusion of multiple numerical and categorical covariates (as in our second example: *hippocampal subfield volumes ~ covariates* + *SNP_i_*). In addition, regarding (i), there is a growing interest in mapping context-dependent genetic effects on both organismal and molecular traits (e.g. interaction effects with environment/exposures [Young, A. *Nat. Commun.* 2017](https://doi.org/10.1038/ncomms12724), tissue/cell type [Kim-Hellmuth et al. *Science* 2020](https://doi.org/10.1126/science.aaz8528), sex/age

[M.B. Sleiman, *Science*, 2022](https://doi.org/10.1126/science.abo3191), etc.), so we believe that the example is timely.

2.3. Finally, while computational time and resource usage is discussed, I think this could benefit from some context as it is not clear to me this is a big problem for GWAS studies. BOLT-LMM and GEMMA have been run on the whole of UK Biobank, so if there is a computational hurdle, it doesn't seem insurmountable. Is this not true for multivariate analysis of sufficient phenotypes?

As the reviewer points out at the end of this comment, besides sample size, the key aspect that impacts running time (and memory usage) of multivariate methods is the number of traits analyzed. Before discussing further, let’s distinguish between three scenarios: i) *univariate* GWAS; ii) *simultaneous univariate* GWAS of multiple traits (i.e. analyzing several traits at the same time thanks to an efficient algorithm able to reuse some of the calculations, which avoids having to analyze the traits sequentially and saves time and resources); and iii) joint, *multivariate* GWAS of several traits (i.e. leveraging the correlation structure of multiple traits to identify associations with the trait vector). Our context is scenario (iii). To the best of our knowledge, BOLT-LMM does not perform multivariate GWAS, just univariate tests (i). As

regards GEMMA, this method does implement both univariate (i) and multivariate (iii) tests. Please note that in our manuscript all our analyses refer to the multivariate version of GEMMA (see *Methods* section lines 228-229).

For both BOLT-LMM and univariate GEMMA, running time and memory usage metrics as a function of the number of individuals (n) have been reported (see e.g. Fig. 1 from [Loh et al,](https://doi.org/10.1038/ng.3190) [*Nat. Genet.*, 2015](https://doi.org/10.1038/ng.3190), or algorithm complexities depicted in Table 1 from [Zhou et al, *Nat.*](https://doi.org/10.1038/s41588-018-0184-y) [*Genet.*, 2018](https://doi.org/10.1038/s41588-018-0184-y)). BOLT-LMM’s running time and memory usage increase roughly as a function of n, while for univariate GEMMA, it increases as a function of n^2^. For the univariate analysis of biobank-scale data, BOLT-LMM is substantially faster than GEMMA, and more widely used. In the case of multivariate GEMMA, although slower than its univariate counterpart, we have found relatively similar results when analyzing few (i.e. 3) traits (see Fig. 2e and Supp. Fig. 17b in our manuscript). This is also in line with what was originally reported by multivariate GEMMA’s authors (see time complexities in Table 1 in [Zhou & Stephens, *Nat.*](https://doi.org/10.1038/nmeth.2848) [*Genet.* 2014](https://doi.org/10.1038/nmeth.2848)).

However, this changes drastically as the number of traits increases (Supp. Fig. 17a). Running time increases at least cubically with the number of traits, q. For a practical example, consider our scenario where n = 1,000 and 10,000 SNPs are tested (Supp. Fig. 17a). In this case, we observed that analyzing 3 traits with GEMMA requires around 1 min, but analyzing 20 traits requires up to 16h. Multivariate GEMMA’s authors have even reported time complexities as a function of q^6^ (see Table 1 in [Zhou & Stephens, *Nat. Genet.* 2014](https://doi.org/10.1038/nmeth.2848), they call d=q). This contrasts strongly with the behaviour of MANTA, for which analyzing either 3 or 20 traits requires only ~1 min. An even more marked difference is observed when looking at memory usage (Supp. Fig. 17c). MANTA’s running time and memory usage increase linearly with respect to n, and sublinearly with respect to q. This is a huge difference with respect to multivariate GEMMA.

Performing multivariate GWAS with GEMMA and other mvLMM methods, based on similar algorithms, is practically infeasible with a moderate/large number of traits at biobank scale. Multivariate GEMMA’s authors indeed recommend to restrict the analyses to a few traits in not very large cohorts: ‘*Our work provides a practical approach to computing LRTs for mvLMMs in reasonably large GWAS (e.g., 50,000 individuals) and a modest number of phenotypes (e.g., 2–10 phenotypes).*’ ([Zhou & Stephens, Nat. Methods, 2014](https://doi.org/10.1038/nmeth.2848)) Even in our example (hippocampal subfield GWAS with 12 traits and around 42,000 individuals, which is not even a very large GWAS), running multivariate GEMMA will not be feasible in practice (see point 2.1 above; even when downsampling the cohort to 10,000 individuals, GEMMA was running for 6 days, which contrasts to the few hours required by MANTA). Finally, GEMMA presents another limitation that precludes its usage in some multivariate scenarios: as we point out in our manuscript, GEMMA’s numerical optimization fails in some situations, particularly when the trait distribution deviates substantially from multivariate normality (as it is the case, for instance of proportion data) or the number of traits analyzed is relatively large (see Results section, lines 212-216 and Methods section, lines 233-236).

In short, I found this a well written and interesting paper. I would like to see how this method compares on real world data

**Second round of review**

**Reviewer 1**

I would like to thank the authors for performing all the additional analysis that I had suggested to better explore the potential biases in the pb-sQTL results. This extensive new analyses are convincing and good additions to the supp materials.

I have no further concerns.

**Reviewer 2**

The authors have done a considerable amount of work and fully answered my comments.
